# Functional Polymorphisms in Xenobiotic Metabolizing Enzymes and Their Impact on the Therapy of Breast Cancer

**DOI:** 10.3389/fgene.2012.00329

**Published:** 2013-01-22

**Authors:** Rosane Vianna-Jorge, Juliana Simões Festa-Vasconcellos, Sheyla Maria Torres Goulart-Citrangulo, Marcelo Sobral Leite

**Affiliations:** ^1^Programa de Farmacologia, Coordenação de Pesquisa, Instituto Nacional do CâncerRio de Janeiro, Brazil; ^2^Instituto de Ciências Biomédicas, Universidade Federal do Rio de JaneiroRio de Janeiro, Brazil

**Keywords:** breast cancer, gene polymorphisms, xenobiotic metabolizing enzymes, hormone therapy, chemotherapy, efficacy, toxicity

## Abstract

Breast cancer is the top cancer among women, and its incidence is increasing worldwide. Although the mortality tends to decrease due to early detection and treatment, there is great variability in the rates of clinical response and survival, which makes breast cancer one of the most appealing targets for pharmacogenomic studies. The recognition that functional *CYP2D6* polymorphisms affect tamoxifen pharmacokinetics has motivated the attempts of using *CYP2D6* genotyping for predicting breast cancer outcomes. In addition to tamoxifen, the chemotherapy of breast cancer includes combinations of cytotoxic drugs, which are substrates for various xenobiotic metabolizing enzymes. Because of these drugs’ narrow therapeutic window, it has been postulated that impaired biotransformation could lead to increased toxicity. In the present review, we performed a systematic search of all published data exploring associations between polymorphisms in xenobiotic metabolizing enzymes and clinical outcomes of breast cancer. We retrieved 43 original articles involving either tamoxifen or other chemotherapeutic protocols, and compiled all information regarding response or toxicity. The data indicate that, although *CYP2D6* polymorphisms can indeed modify tamoxifen pharmacokinetics, *CYP2D6* genotyping alone is not enough for predicting breast cancer outcomes. The studies involving other chemotherapeutic protocols explored a great diversity of pharmacogenetic targets, but the number of studies for each functional polymorphism is still very limited, with usually no confirmation of positive associations. In conclusion, the application of pharmacogenetics to predict breast cancer outcomes and to select one individual’s chemotherapeutic protocol is still far from clinical routine. Although some very interesting results have been produced, no clear practical recommendations are recognized yet.

## Introduction

Breast cancer is the most frequent type of cancer in women (Jemal et al., 2011), and the second leading cause of cancer-related death in women worldwide (DeSantis et al., 2011). The incidence rates of breast cancer are increasing both in developed and in developing countries (Ferlay et al., 2010), whereas the mortality rates have decreased in the last decade [World Health Organization (WHO), 2004; Ferlay et al., 2010; DeSantis et al., 2011; Jemal et al., 2011], probably because of the investments in early detection and in new pharmacological approaches (Berry et al., 2005). Although the recent decrease in the mortality rates proves the efficacy of the current therapeutic protocols, the optimization of available therapies is crucial. Most anticancer drugs are highly toxic, and many patients suffer with adverse reactions that might be persistent throughout the treatment, and sometimes even irreversible. In addition to their obvious impact in patients’ quality of life, drug toxicities may also require dose delays, treatment modifications, or even treatment interruption, contributing to the great variability that is usually observed in breast cancer clinical outcomes. This scenario makes breast cancer one of the most appealing targets for evaluation of pharmacogenomic strategies toward personalized medicine.

The current treatment of breast cancer consists of combinations of surgery and adjuvant or neoadjuvant therapeutic approaches, including radiotherapy, cytotoxic chemotherapy, hormonal therapy, and targeted therapy. The treatment choice is routinely based on the estimated risk of recurrence, considering the clinical stage at diagnosis and molecular predictive factors (Soerjomataram et al., 2008). Nevertheless, breast cancer is a very heterogeneous disease, with a continuous grading in tumor histology (Hayes et al., 2001), different cellular origins (Anderson and Matsuno, 2006), and great molecular diversity (Danova et al., 2011), which make prognostic estimates a difficult task, especially in early-stage tumors. In an attempt to improve the classical pathology-driven classification of breast tumors, a series of efforts are currently in course, including the description of gene expression patterns (Perou et al., 2000; Sørlie et al., 2001), and of genomic signatures (Banerji et al., 2012; Nik-Zainal et al., 2012; Stephens et al., 2012). In addition to molecular variations in the tumor, the individual genetic diversity may also contribute for the great heterogeneity in treatment outcomes. Thus, inherited sequence variations (polymorphisms) in genes involved in the pharmacokinetics and pharmacodynamics of anticancer drugs may affect both their efficacy and safety (O’Donnell and Ratain, 2012; Ruiz et al., 2012).

The chemotherapy of breast cancer includes different options of drug combinations. Anthracycline-based protocols have become the standard adjuvant and neoadjuvant chemotherapy for most patients in view of clinical evidences of improved efficacy in comparison to other previously used protocols (Hassan et al., 2010). More recently, taxanes, such as docetaxel or paclitaxel, were added to anthracycline-based protocols, further reducing the risk of recurrence (De Laurentiis et al., 2008; Martín et al., 2010; Jacquin et al., 2012). The most usual protocols for breast cancer chemotherapy nowadays are: docetaxel, doxorubicin, and cyclophosphamide (TAC); docetaxel, epirubicin, and cyclophosphamide (TEC); cyclophosphamide, doxorubicin, and 5-fluorouracil, followed by docetaxel (CAF-T); doxorubicin and cyclophosphamide (AC); and doxorubicin and cyclophosphamide, followed by paclitaxel or docetaxel (AC-P or AC-T). The older protocol cyclophosphamide, methotrexate, and 5-fluorouracil (CMF) is also still used.

Cytotoxic antineoplastic drugs have narrow therapeutic window, and small variations in their plasma concentrations may lead to clinically significant toxicity. Taxanes, for example, may cause severe bone marrow dysfunction, and their toxic effects present great interpatient variability, which appear to be due to interindividual differences in pharmacokinetic parameters (Engels et al., 2011). Accordingly, the biotransformation of taxanes is mainly mediated by hepatic CYP450s, which may occur in different isoforms, with distinct functional activities, as a consequence of polymorphisms in their coding genes. Doxorubicin and epirubicin may cause severe cardiotoxicity (Doyle et al., 2005; Pinder et al., 2007; Gianni et al., 2008) and bone marrow dysfunction (Hershman et al., 2007; Patt et al., 2007). Their biotransformation includes reductions by carbonyl reductases (CBR1 and CBR3) and by aldoketoreductases (AKR1A1 and AKR1C3; Lal et al., 2010). Epirubicin also undergoes conjugation by uridine diphosphate-glucuronosyltransferase 2B7 (UGT2B7; Innocenti et al., 2001). Finally, glutathione (*S*)-transferases (GSTs) may also participate to detoxification. Like the CYP450s, all these xenobiotic metabolizing enzymes are coded by polymorphic genes, which make them potential targets for pharmacogenomic evaluations.

In addition to the cytotoxic antineoplastic drugs, tamoxifen is perhaps the most appealing target for breast cancer pharmacogenomics. It was approved by the FDA in 1977, and since then, it is still the drug with the most striking effect on patients’ survival, reducing the annual risk of recurrence by 39% after a 5-year treatment [Early Breast Cancer Trialists Collaborative Group (EBCTCG), 2005]. The antitumor effects of tamoxifen are mediated by selective modulation of the estrogen receptor, which can be detected in more than two thirds of breast tumors, and to consequent inhibition of estrogen-dependent cell proliferation. However, in spite of the undisputable efficacy and long-term benefits of tamoxifen for breast cancer patients, there is great interindividual variability in the degree of response. Thus, approximately half of the estrogen receptor-positive tumors do not respond to tamoxifen therapy (Jaiyesimi et al., 1995; Osborne, 1998; Buzdar, 2001), and the 15-year recurrence probability in early breast cancer patients treated with tamoxifen for 5 years is approximately one third [Early Breast Cancer Trialists Collaborative Group (EBCTCG), 2005].

The great interindividual variability observed in the degree of response to tamoxifen can be ascribed to different causes, including failures in patient adherence, drug interactions, and genetic variations affecting tamoxifen pharmacokinetics (Hoskins et al., 2009). This variability is of special concern in premenopausal women, who cannot receive aromatase inhibitors, and therefore have less therapeutic options. Thus, it seems crucial to find strategies to ensure tamoxifen response, minimize, or predict individual variability, and improve disease outcomes. Besides the interpatient variability in the degree of response, another motivation for pharmacogenomic studies would be tamoxifen safety. The main concern refers to the risk of endometrial cancer and of thromboembolic events, although these are quite rare events (Fisher et al., 1998). The most common side effect of tamoxifen therapy is hot flushes, which are intrinsically correlated with tamoxifen’s antiestrogenic activity. Although hot flushes do not represent a life threat, and might become more tolerable with therapy continuation, they can be so intense that patients stop tamoxifen use.

Tamoxifen is considered a prodrug, with very little affinity for the estrogen receptor in its original structure (Coezy et al., 1982). The pharmacological actions of tamoxifen are most likely due to its metabolites (Coezy et al., 1982; Robertson et al., 1982), which are generated in the liver by numerous phase I and II reactions (Mürdter et al., 2011). The major metabolite is *N*-desmethyl-tamoxifen, which is generated by CYP3A4/5 and accounts for approximately 90% of tamoxifen metabolites (Desta et al., 2004). However, *N*-desmethyl-tamoxifen shows little affinity for the estrogen receptor when compared to two other metabolites, 4-hydroxy-tamoxifen and 4-hydroxy-*N*-desmethyl-tamoxifen (endoxifen; Coezy et al., 1982; Jordan, 1982; Robertson et al., 1982). Because 4-hydroxy-tamoxifen and endoxifen have similar potencies in suppressing estrogen-dependent cell proliferation, but the latter appears to be generated in higher concentrations, endoxifen is believed to be the major active metabolite *in vivo* (Johnson et al., 2004; Lim et al., 2005). Endoxifen generation is mainly dependent on CYP2D6 activity (Desta et al., 2004), and initial observations suggested that genetic polymorphisms in its coding gene, *CYP2D6*, could affect the activity of CYP2D6, resulting in reduced plasma level of endoxifen (Jin et al., 2005). Corroborating this notion, *CYP2D6* polymorphisms responsible for reduced CYP2D6 activity were associated with worse breast cancer outcomes in postmenopausal estrogen receptor-positive patients treated with tamoxifen (Goetz et al., 2005). These results prompted the FDA to recommend, in 2006, an update in the tamoxifen package insert, alerting for the increased risk of breast cancer recurrence in patients who are CYP2D6 poor metabolizers.

The awareness of the potential impact of *CYP2D6* polymorphisms in tamoxifen pharmacokinetics and pharmacodynamics has motivated a series of pharmacogenomic studies, designed to explore the possibility of using *CYP2D6* genotyping for predicting clinical outcomes in breast cancer patients receiving tamoxifen. According to more recent data, it seems clear that genetic polymorphisms that modulate CYP2D6 activity can indeed modify endoxifen plasma levels (Kiyotani et al., 2010; de Graan et al., 2011; Irvin et al., 2011; Lim et al., 2011; Mürdter et al., 2011). However, the impact of such pharmacokinetic changes on the individual degree of response to tamoxifen is less clear, and *CYP2D6* genotyping alone has been not enough for predicting breast cancer outcomes in clinical settings (Abraham et al., 2010; Rae, 2011; Regan et al., 2012). It appears, thus, that the pharmacological actions of tamoxifen may be more complex than initially thought, with its antiestrogenic activity being dependent not on a single metabolite, but on a composite action of them (Rae et al., 2011). As a consequence of this new assumption, other genetic polymorphisms affecting the pharmacokinetics of tamoxifen might have additional influences on breast cancer outcomes, and should also be considered in pharmacogenomic studies. Likewise, there might be combined influences of genetic polymorphisms on the pharmacokinetics/dynamics of both tamoxifen and other cytotoxic chemotherapeutic drugs, which would require much more complex study designs for evaluation of their impact in clinical settings.

In the present review, we aimed to compile all information available on pharmacogenomic studies involving polymorphisms in xenobiotic metabolizing enzymes and their consequences in clinical outcomes of breast cancer. The analyzed studies explore either tamoxifen or the antineoplastics used in chemotherapy. Instead of focusing on selected positive associations, we performed a systematic review of all published data, evaluating the reported effects on both response and toxicity, as well as all the recorded information of null associations.

## Materials and Methods

A systematic review of the literature data was conducted, which was performed via electronic search of the MEDLINE database (available at PUBMED), and included articles available until August 2012. The search terms were selected using the controlled vocabulary MeSH for the PubMed database. The main search was as follows: [“polymorphism, genetic” (MeSH Terms)] AND [“breast neoplasms” (MeSH Terms)] AND [enzyme (Text Word) OR “enzymes” (MeSH Terms)] AND [breast cancer (Title/Abstract)] AND (xenobiotic OR drug) NOT [“Review” (Publication Type)]. The following filters were used: Published in the last 10 years; Humans; English; Female.

All abstracts were retrieved, and were used for selection of articles to be used in the review. The pre-defined inclusion criteria were: original articles, including clinical trials, prospective, or retrospective observational studies, describing correlations between polymorphisms in xenobiotic metabolizing enzymes and any clinical response or outcome of breast cancer patients under therapeutic treatment. The exclusion criteria were: letters, commentaries, editorials, or case reports; studies involving only the susceptibility of developing cancer; studies involving only drug transporters or other enzymes not responsible for xenobiotic metabolism; studies involving only pharmacokinetic analyses or correlation with histopathological features, without evaluation of clinical outcomes.

Two reviewers performed the selection of articles to be included in the review according to previously defined inclusion and exclusion criteria, and extracted information for data compilation. A third reviewer examined the lists of selected and excluded articles in order to confirm the eligibility, and checked all the extracted information.

The compiled information was evaluated separately for tamoxifen and for other chemotherapeutic protocols, and focused on the associations between polymorphisms and drug efficacy or drug safety. All the null associations were also collected, compiled, and analyzed.

## Results

**Figure 1:**
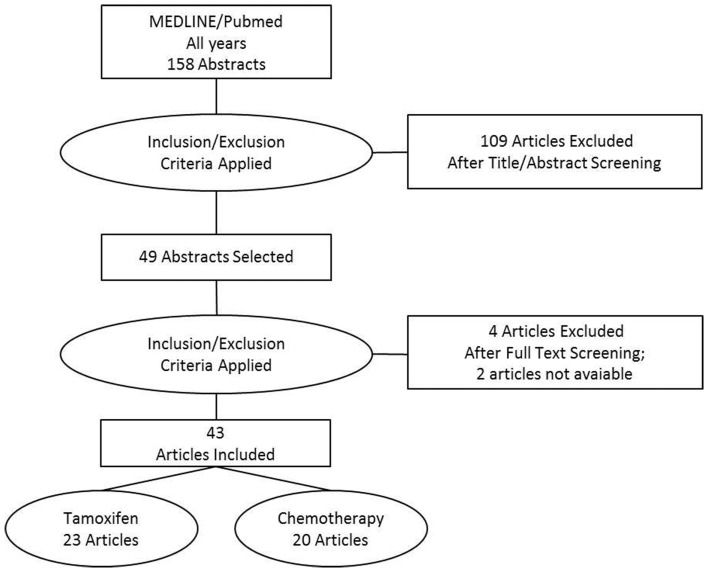
**Flow chart of the systematic review of literature data**.

The literature search resulted in 158 abstracts among the bibliographic references from PUBMED. The abstract reading led to the selection of 30 original articles involving tamoxifen use, and 22 original articles involving any kind of breast cancer chemotherapy. The excluded items (106) consisted of non-original articles (4), articles not involving xenobiotic metabolizing enzymes (85), articles evaluating only the susceptibility to breast cancer (10), articles evaluating only the correlation with histopathological features (3), articles involving only pharmacokinetic analyses (2), 1 article evaluating clinical outcomes not related to breast cancer treatment, and 1 article not involving humans. Among the 49 selected abstracts, 2 articles could not be retrieved, and 7 articles did not fit the inclusion criteria. The excluded articles after full-text reading were: 2 articles not involving xenobiotic metabolizing enzymes, 1 article based on previously published data, 1 article analyzing breast cancer patients together with other cancer patients, 1 article involving only pharmacokinetic analyses, 1 article evaluating clinical outcomes not related to breast cancer treatment, and 1 article which was retracted. The selected articles consisted of 23 original studies involving tamoxifen use, and 20 original studies involving other chemotherapeutic protocols (Figure 1).

The selected studies were mostly based on prospective, observational designs, and focused on the evaluation of different breast cancer outcomes, defined either as primary or secondary endpoints. Some studies also included pharmacokinetic evaluations or other endpoints not directly related to breast cancer treatment. The extracted information was restricted to polymorphisms in xenobiotic metabolizing enzymes and to any clinical outcome directly correlated to breast cancer treatment. The results are distributed in five Tables: Tables 1 and 2 present the compiled data involving tamoxifen treatment, and comprise results on both efficacy and safety. Table 1 presents only statistically significant associations, whereas Table 2 presents only null associations. Tables 3– 5 present the compiled data involving any adjuvant, neoadjuvant, or palliative chemotherapeutic protocol. Table 3 presents the significant associations involving efficacy outcomes, Table 4 presents any significant association involving toxicities, and Table 5 presents all reported null associations, involving either efficacy or safety outcomes.

**Table 1:** **Effects of polymorphisms in xenobiotic metabolizing enzymes on breast cancer outcomes after tamoxifen treatment**.

Gene	SNP	Reference	Population	Design	*N*	Reference group^a^	Affected group^b^	Outcome	Main results	
									Calculation (95% CI)	*p*-Value
*CYP2D6*	*4	Goetz et al. (2005)	Adjuvant	Cohort	223	*1/*1	*4/*4	RFT	KM–LR^c^	0.03
						*1/*1	*4/*4	DFS	KM–LR^c^	0.02
		Wegman et al. (2007)	Adjuvant	Cohort	677	*4/*4	*1/*4	RFS	KM–LR^c^	0.04
		Bijl et al. (2009)	All incident users	Cohort	85	*1/*1	*4/*4	BCSS	HR; 4.1 (1.1–15.9)	0.041
						*1/*1	*1/*4 or *4/*4	BCSS	HR; 2.1 (1.1–4.2)	0.031
						*1	*4	BCSS	HR; 2.0 (1.1–3.4)	0.015
	*6	Abraham et al. (2010)	Adjuvant	Case-cohort	3155	*1/*1	*1/*6	BCSS	HR; 1.95 (1.1–3.4)	0.02
	*10	Lim et al. (2007)	Adjuvant, palliative	Cohort	202	*1/*1	*10/*10	TTP	KM–LR^c^	0.0032
		Xu et al. (2008)	Adjuvant	Cohort	152	*1/*1	*10/*10	5-Years DFS	HR; 4.7 (1.1–20.0)	0.04
**COMBINED *CYP2D6* GENOTYPES**										
		Newman et al. (2008)^d^	Adjuvant	Cohort	115	EM or Het-IM	IM	RFS	KM–LR^c^	0.031
						*1/*1	PM	TTR	HR; 2.9 (0.9–9.4)	0.076
						*1/*1	PM	OS	HR; 3.5 (0.8–15.4)	0.079
		Schroth et al. (2009)^e^	Adjuvant	Retrospective cohort	1325	EM	PM	TTR	HR; 1.90 (1.10–3.3)	0.02
		Schroth et al. (2010)^f^	Adjuvant	Cohort	492	EM	PM	RFS	HR; 2.77 (1.3–5.9)	0.011^g^
		Kiyotani et al. (2010)^h^	Adjuvant	Cohort	282	EM	Het-EM/IM	TTR	HR; 1.40 (1.0–1.9)	0.03
						*1/*1	*1/V	RFS	HR; 4.44 (1.3–15.0)	0.000036^g^
						*1/*1	*V/V*	RFS	HR; 9.52 (2.8–32.4)	0.000036^g^
		Teh et al. (2012)^i^	Non-palliative	Cohort	95	EM	IM	RFS	OR; 13.14 (1.54–109.9)	0.004
*CYP2C8*	*3	Jernström et al. (2009)	Adjuvant	Cohort	297	*1/*1	*1/*3 or *3/*3	BCSS	HR; 9.10 (1.4–59.6)	0.021
						*1/*1	*1/*3 or *3/*3	DFS	HR; 8.56 (1.5–1.1)	0.015
*CYP2C8*–*CYP2C9*	*3–*2	Jernström et al. (2009)	Adjuvant	Cohort	297	*1/*1	*1/*3–*1/*2	DFS	HR; 2.54 (1.5–5.79)	0.015
*CYP2C19*	*2	Ruiter et al. (2010)	All incident users	Cohort	80	*1/*1	*2 Carriers	BCSS	HR; 0.26 (0.08–0.9)	0.03
		van Schaik et al. (2011)	Palliative	Cohort	499	*1/*1	*2 Carriers	TTF	HR; 0.72 (0.57–0.90)	0.04
	*17	van Schaik et al. (2011)	Palliative	Cohort	499	*1/*1	*17 Carriers	DFS	HR; 0.66 (0.46–0.95)	0.025
*CYP3A5*	*3	Wegman et al. (2007)	Adjuvant	Cohort	677	*1/*1 or *1/*3	*3/*3	RFS	HR; 0.13 (0.02–0.9)	0.03
*SULT1A1*	*2	Nowell et al. (2002)	Adjuvant	Cohort	160	*1/*1 or *1/*2	*2/*2	OS	HR; 2.9 (1.1–7.6)	
		Wegman et al. (2007)	Adjuvant	Cohort	677	*1/*2 or *2/*2	*1/*1	RFS (2 years TAM)	HR; 0.33 (0.1–0.96)	0.04
**COMBINED GENOTYPES**										
*SULT1A1* and *UGT2B15*		Nowell et al. (2005)	Adjuvant	Case-cohort	162	SULT1A1 (*1/*1 or *1/*2), UGT2B15*1/*1	SULT1A1*2/*2, UGT2B15 (*1/*2 or *2/*2)	OS	HR; 4.40 (1.2–16.5)	0.03^g^
						SULT1A1 (*1/*1 or *1/*2), UGT2B15*1/*2	SULT1A1*2/*2, UGT2B15 (*1/*2 or *2/*2	PFS	HR; 3.79 (1.2–12.1)	0.03^g^
*CYP2D6* and *CYP2C19*		van Schaik et al. (2011)	Palliative	Cohort	499	CYP2D6*1/*1, CYP2C19 *1/*1	CYP2D6 (*1/*4 or *4/*4), CYP2C19 (*1/*17 or *17/*17)	TTF	KM–LR^c^	0.031

BCSS, breast cancer-specific survival; DFS, disease-free survival; TTF, time-to-treatment failure; EM, extensive metabolizer; Het, heterozygous; Homo, homozygous; HR, hazard ratio; IM, intermediate metabolizer; KM–LR, Kaplan–Meier curves and Log-Rank test; OR, odds ratio; OS, overall survival; PFS, progression-free survival; PM, poor metabolizer; RFS, recurrence-free survival; RFT, relapse-free time; RR, risk relative; TAM, tamoxifen; TTP, time to progression; TTR, time to recurrence; V, variant alleles. ^a^The Reference group includes alleles, genotypes, haplotypes, or phenotypes considered as reference for evaluation of outcome parameters. The designation *1 indicates the reference allele sequence for the analyzed SNP, but may include non-analyzed sequence variations. ^b^The Affected group includes alleles, genotypes, haplotypes, or phenotypes with significantly different outcome parameters, when compared to the Reference group. ^c^The Affected group had a worse outcome. ^d^Newman et al. (2008) – PM: two copies of the CYP2D6*3, CYP2D6*4, or CYP2D6*5 alleles; a group with concomitant use of a potent CYP2D6 inhibitor in wild-type individuals or moderate inhibitor in heterozygous patients. ^e^Schroth et al. (2009) – PM: presence of two alleles *3, *4, or *5; EM: absence of alleles *3, *4, *5, *10, and *41; Het-EM/IM: presence of two alleles *10 or *41, or presence of only one allele *3, *4, or *5; ^f^Schroth et al. (2010) – EM: presence of alleles *1, *2, or *35; PM: presence of two alleles *3, *4, *5, *6, *7, or *8; ^g^Ptrend values. ^h^Kiyotani et al. (2010) – V: *4, *5, *10, *14, *21, *36, or *41; ^i^Teh et al. (2012) – EM: absence of alleles *xN, *4, *5, *10, and *14; Het-IM: *1/*10; IM: *10/*10 or heterozygous null alleles (*1/*4, *1/*5, *1/*14).

**Table 2:** **Null associations between polymorphisms in xenobiotic metabolizing enzymes and breast cancer outcomes after tamoxifen treatment**.

Gene	SNP	Reference	Population	Design	*N*	Compared groups^a^			Outcome
*CYP2D6*	*4	Goetz et al. (2005)	Adjuvant	Cohort	223	*1/*1 or *1/*4	vs.	*4/*4	RFS
						*1/*1 or *1/*4	vs.	*4/*4	DFS
						*1/*1 or *1/*4	vs.	*4/*4	Hot flushes
		Nowell et al. (2005)	Adjuvant	Case-cohort	162	*1/*1	vs.	*4 Carrier	PFS
		Wegman et al. (2005)	Adjuvant	Cohort	112	*1/*1	vs.	*1/*4 or *4/*4	RFS
		Wegman et al. (2007)	Adjuvant	Cohort	677	*1/*1	vs.	*4/*4	RFS
						*1/*1	vs.	*1/*4 or *4/*4	RFS
		Abraham et al. (2010)	Adjuvant	Case-cohort	3155	*1/*1	vs.	*4 Carrier	BCSS
		Regan et al. (2012)	Adjuvant	Cohort	4393	*1/*1	vs.	*4/*4	BCFI
						*1/*1	vs.	*1/*4	BCFI
		van Schaik et al. (2011)	Palliative	Cohort	499	*1/*1	vs.	*4 Carrier	TTF
	*5	Abraham et al. (2010)	Adjuvant	Case-cohort	3155	*1/*1	vs.	*5 Carrier	BCSS
	*9	Abraham et al. (2010)	Adjuvant	Case-cohort	3155	*1/*1	vs.	*9 Carrier	BCSS
	*10	Okishiro et al. (2009)	Adjuvant	Cohort	173	*1/*1 or *1/*10	vs.	*10/*10	RFS
						*1/*1 or *1/*10	vs.	*10/*10	Bone mineral density
						*1/*1 or *1/*10	vs.	*10/*10	Total cholesterol
						*1/*1 or *1/*10	vs.	*10/*10	Endometrial thickness (1 year TAM)
		Toyama et al. (2009)	Adjuvant	Cohort	156	*1/*1	vs.	*1/*10 or *10/*10	DFS
						*1/*1	vs.	*1/*10 or *10/*10	DFS
						*1/*1	vs.	*1/*10 or *10/*10	BCSS
		Abraham et al. (2010)	Adjuvant	Case-cohort	3155	*1/*1	vs.	*10 Carrier	BCSS
		Xu et al. (2008)	Adjuvant	Cohort	152	*1/*1	vs.	*10 Carrier	5-Year DSS
	*41	Abraham et al. (2010)	Adjuvant	Case-cohort	3155	*1/*1	vs.	*41 Carrier	BCSS
Combined *CYP2D6* genotypes		Abraham et al. (2010)^b^	Adjuvant	Case-cohort	3155	*1/*1	vs.	PM or IM	BCSS
						*1/*1	vs.	PM	BCSS
		Regan et al. (2012)^d^	Adjuvant	Cohort	4393	EM	vs.	PM	BCFI
						EM	vs.	IM	BCFI
						EM	vs.	PM or IM	BCFI
						EM	vs.	PM	Hot flushes
						EM	vs.	IM	Hot flushes
						EM	vs.	PM/IM	Hot flushes
		Teh et al. (2012)^c^	Non-palliative	Cohort	95	EM	vs.	Het-IM	RFS
		Newman et al. (2008)^e^	Adjuvant	Cohort	115	*1/*1	vs.	PM	TTR
						*1/*1	vs.	PM	OS
*CYP2C8*	*4	Jernström et al. (2009)	Adjuvant	Cohort	297	*1/*1	vs.	*1/*4 or *4/*4	BCSS
*CYP2C9*	*2	Jernström et al. (2009)	Adjuvant	Cohort	297	*1/*1	vs.	*1/*2 or *2/*2	BCSS
	*3					*1/*1	vs.	*1/*3 or *3/*3	BCSS
*CYP2C19*	*2 and *3	Okishiro et al. (2009)	Adjuvant	Cohort	173	*1/*1, *1/*2, or *1/*3	vs.	*2/*2, *2/*3, or *3/*3	RFS
						*1/*1, *1/*2, or *1/*3	vs.	*2/*2, *2/*3, or *3/*3	Bone mineral density
						*1/*1, *1/*2, or *1/*3	vs.	*2/*2, *2/*3, or *3/*3	Total cholesterol
						*1/*1, *1/*2, or *1/*3	vs.	*2/*2, *2/*3, or *3/*3	Endometrial thickness (1 year TAM)
	*2	van Schaik et al. (2011)	Palliative	Cohort	499	*1/*1	vs.	*2 Carrier	DFS
	*17	Moyer et al. (2011)	All incident users	Cohort	190	*1/*1	vs.	*1/*17 or *17/*17	DFS
		van Schaik et al. (2011)	Palliative	Cohort	499	*1/*1	vs.	*17 Carrier	TTF
*CYP3A5*	*3	Goetz et al. (2005)	Adjuvant	Cohort	223	*1/*1 or *1/*3	vs.	*3/*3	RFS
						*1/*1 or *1/*3	vs.	*3/*3	DFS
						*1/*1 or *1/*3	vs.	*3/*3	Hot flushes
		Tucker et al. (2005)	Adjuvant	Cohort	98	*1/*1	vs.	*1/*3 or *3/*3	Nausea
						*1/*1	vs.	*1/*3 or *3/*3	Migraines
						*1/*1	vs.	*1/*3 or *3/*3	Depression
						*1/*1	vs.	*1/*3 or *3/*3	Vaginal discharge
						*1/*1	vs.	*1/*3 or *3/*3	Vaginal dryness
						*1/*1	vs.	*1/*3 or *3/*3	Insomnia
						*1/*1	vs.	*1/*3 or *3/*3	Hot flushes
	*6	Tucker et al. (2005)	Adjuvant	Cohort	98	*1/*1	vs.	*1/*6 or *6/*6	Nausea
						*1/*1	vs.	*1/*6 or *6/*6	Migraines
						*1/*1	vs.	*1/*6 or *6/*6	Depression
						*1/*1	vs.	*1/*6 or *6/*6	Vaginal discharge
						*1/*1	vs.	*1/*6 or *6/*6	Vaginal dryness
						*1/*1	vs.	*1/*6 or *6/*6	Insomnia
						*1/*1	vs.	*1/*6 or *6/*6	Hot flushes
*SULT1A1*	*2	Wegman et al. (2005)	Adjuvant	Cohort	112	*1/*1	vs.	*1/*2 or *2/*2	RFS
		Wegman et al. (2007)	Adjuvant	Cohort	677	*1/*1	vs.	*1/*2 or *2/*2	RFS
	Copy number	Moyer et al. (2011)	All incident users	Cohort	190	≤*2*	vs.	>*2*	DFS
*UGT1A8*	*3	Ahern et al. (2011)	Non-palliative	Case-cohort	541	*1/*1	vs.	*1/*3 or *3/*3	RFS
*UGT2B7*	*2	Ahern et al. (2011)	Non-palliative	Case-cohort	541	*1/*1	vs.	*2/*2	RFS
*UGT2B15*	*2	Nowell et al. (2005)	Adjuvant	Case-cohort	162	*1/*1	vs.	*1/*2	RFS
						*1/*1	vs.	*2/*2	
		Wegman et al. (2007)	Adjuvant	Cohort	677	*1/*1	vs.	*1/*2, *2/*2	RFS
		Ahern et al. (2011)	Non-palliative	Case-cohort	541	*1/*1	vs.	*2/*2	RFS

Abbreviations: BCFI, breast cancer-free interval; BCSS, breast cancer-specific survival; DFS, disease-free survival; DSS, disease-specific survival; EM, extensive metabolizer; Het, heterozygous; Homo, homozygous; IM, intermediate metabolizer; PFS, progression-free survival; PM, poor metabolizer; RFS, recurrence-free survival; TTF, time-to-treatment failure; V, variant alleles. ^a^The designation *1 indicates the reference allele sequence for the analyzed SNP, but may include non-analyzed sequence variations. ^b^Abraham et al. (2010) – PM: presence of at least one allele *4, *5, or *6; IM: presence of one or two alleles *9, *10, or *41; ^c^Teh et al. (2012) – EM: absence of alleles *xN, *4, *5, *10, and *14; Het-IM: *1/*10; IM: *10/*10 or heterozygous null alleles (*1/*4, *1/*5, *1/*14); ^d^Regan et al. (2012) – PM: presence of alleles *3, *4, *6, or *7; IM: *41/*41 or *41/PM; ^e^Newman et al. (2008) – PM: two copies of the CYP2D6*3, CYP2D6*4, or CYP2D6*5 alleles; a group with concomitant use of a potent CYP2D6 inhibitor in wild-type individuals or moderate inhibitor in heterozygous patients.

**Table 3:** **Effects of polymorphisms in xenobiotic metabolizing enzymes on breast cancer outcomes after chemotherapy**.

Gene	SNP	Reference	Population/design	Protocol	*N*	Reference group^a^	Affected group^b^	Outcome	Main results	
									Calculation (95% CI)	*p*-Value
*CYP1A1*	*m2*	Chacko et al. (2005)	Adjuvant, neoadjuvant, metastatic	Not informed	79	*wt/wt*	*wt/m2* or *m2/m2*	BCSS	HR; 18.3 (2.4–140)	0.005
*CYP1B1*	*3	Marsh et al. (2007)	Stages IIIA – IV	Paclitaxel-based	84	*1/*1 or *1/*3	*3/*3	PFS	KM–LR^c^	0.037
*CYP2B6*	*2	Bray et al. (2010)	Adjuvant	AC	230	*1/*1	*1/*2	TTP	KM–LR^c^	0.002
	*4	Bray et al. (2010)	Adjuvant	AC	230	*1/*1	*4/*4	OS	KM–LR^c^	0.05
	*9	Bray et al. (2010)	Adjuvant	AC	230	*1/*1	*9/*9	OS	KM–LR^c^	0.003
*CYP3A4*	*1B	Gor et al. (2010)	Adjuvant	CAF (+ CTX/thiotepa)	350	*1B/*1A	*1A/*1A	DFS	HR; 2.79 (1.5–5.1)	0.001
*GSTA1*	*B	Sweeney et al. (2003)	Neoadjuvant	CTX-based		*A/*A or *A/*B	*B/*B	OS	HR; 0.5 (0.3–0.8)	0.01
*GSTP1*	*313A* > *G*	Huang et al. (2008)	Adjuvant	FEC	192	*A/A (Ile/Ile)*	*A/G (Ile/Val) or G/G (Val/Val)*	Early relapse^d^	χ^2^ Test^c,e^	0.014
	*313A* > *G*	Zhang et al. (2011)	Neoadjuvant	EPI and CTX	120	*G/G (Val/Val)*	*A/A (Ile/Ile) or A/G (Ile/Val)*	Pathological response	OR; 0.4 (0.2–0.96)	0.024
*GSTT1*	Deletion (null)	Chacko et al. (2005)	Adjuvant, neoadjuvant, metastatic	Not informed	79	Non-null	Null	BCSS	HR; 0.2 (0.0–0.9)	0.039
*UGT2B7*	*His268Tyr*	Parmar et al. (2011)	Adjuvant	EPI	205	Two *268Tyr* alleles	At least one *268His* allele	DFS	HR; 2.64 (1.2–5.7)	0.014
**COMBINED GENOTYPES**										
*GSTP1/SOD2*	*313A* > *G*	Bewick et al. (2008)	Metastatic/retrospective cohort	MITOX and CTX	95	*GSTP1 AA, and SOD2 CC or CT*	*GSTP1 GG or AG, and SOD2 TT*	BCSS	HR; 2.17 (1.1–4.2)	0.013
	*313A* > *G/16C* > *T*	Bewick et al. (2008)	Metastatic/retrospective cohort	MITOX and CTX	96	*GSTP1 AA, and SOD2 CC or CT*	*GSTP1 GG or AG, and SOD2 TT*	PFS	HR; 2.89 (1.4–5. 9)	0.002

Abbreviations: AC, adriamycin and cyclophosphamide; BCSS, breast cancer-specific survival; CAF, cyclophosphamide, adriamycin, and 5-fluorouracil; CTX, cyclophosphamide; DFS, disease-free survival; EPI, epirubicine; FEC, 5-fluorouracil, epirubicine, and cyclophosphamide; HR, hazard ratio; KM–LR, Kaplan–Meier curves and Log-Rank test; MITOX, mitoxantrone; OR, odds ratio; OS, overall survival; PFS, progression-free survival; RR, relative risk; TEC, docetaxel, epirubicine, and cyclophosphamide; TTP, time to progression. WT, wild-type allele. ^a^The *Reference group* includes alleles, genotypes, haplotypes, or phenotypes considered as reference for evaluation of outcome parameters. The designation *1 indicates the reference allele sequence for the analyzed SNP, but may include non-analyzed sequence variations. ^b^The *Affected group* includes alleles, genotypes, haplotypes, or phenotypes with significantly different outcome parameters, when compared to the *Reference group*. ^c^The *Affected group* had a worse outcome. ^d^Early relapse was designated as the development of new recurrent or distant metastatic lesions within 2 years after receiving postoperative chemotherapy; ^e^χ^2^ test.

**Table 4:** **Effects of polymorphisms in xenobiotic metabolizing enzymes on the toxicity to chemotherapy for breast cancer**.

Gene	SNP	Reference	Population	*N*	Protocol	Outcome	Reference group	Affected group	Main results	
									Calculation (95% CI)	*p*-Value
*CBR3*	*G11A*	Fan et al. (2008)	Neoadjuvant	99	Doxorubicin and docetaxel	Leucopenia at nadir^a^	*GG*	*GA, AA*	Trend test	0.019
						Thrombocytopenia at nadir^a^	*GG*	*GA, AA*	Trend test	0.026
*CYP1B1*	*3	Rizzo et al. (2010)	Adjuvant, neoadjuvant, or metastatic	95	Taxanes	Hypersensitivity^b^	*CG* or *GG*	*CC*	OR; 0.136 (0.05–0.37)	0.014
*CYP2B6*	*2	Bray et al. (2010)	Adjuvant	230	AC	Toxicity and dose delay	*CT* or *TT*	*CC*	χ2 Test^c^	0.013
*CYP3A5*	*3	Tsai et al. (2009)	Adjuvant or neoadjuvant	59	TEC	Febrile neutropenia	*3/*3	*1/*3	RR; 7.17 (1.10–3.55)	*p* < 0.05
		Tsai et al. (2009)	Adjuvant or neoadjuvant	59	TEC	Fever	*3/*3	*1/*3	RR; 3.29 (1.03–10.05)	*p* < 0.05
		Tsai et al. (2009)	Adjuvant or neoadjuvant	59	TEC	Neutropenia	*3/*3	*1/*3	RR; 3.29 (1.03–10.5)	*p* < 0.05
*GSTP1*	*Ile105Val*	Zárate et al. (2007)	Adjuvant	95	Anthracycline-based	Hematological toxicity (grades 3 or 4)	*A/A (Ile/Ile)* or *A/G (Ile/Val)*	*G/G (Val/Val)*	HR; 6.4 (1.05–39)	0.044
		Yao et al. (2010)	Adjuvant	874	CAF or CMF	Neutropenia (grades 3 or 4)	*A/A (Ile/Ile)*	*A/G (Ile/Val)* or *G/G (Val/Val)*	OR; 0.63 (0.41–0.97)	0.04
		Yao et al. (2010)	Adjuvant	874	CAF or CMF	Leucopenia	*A/A (Ile/Ile)*	*A/G (Ile/Val)* or *G/G (Val/Val)*	OR; 0.59 (0.39–0.89)	0.01
		Zhang et al. (2011)	Neoadjuvant	120	EPI and CTX	Severe toxicity (grades 3 or 4)	*G/G (Val/Val)*	*A/A (Ile/Ile) or A/G (Ile/Val)*	OR; 0.35 (0.13–0.78)	0.006

*Abbreviations: AC, driamycin and cyclophosphamide; BCSS, breast cancer-specific survival; CAF, cyclophosphamide, adriamycin, and 5-fluorouracil; CMF, cyclophosphamide, metothrexate, and 5-fluorouracil; CTX, cyclophosphamide; DFS, disease-free survival; EPI, epirubicine; FEC, 5-fluorouracil, epirubicine, and cyclophosphamide; HR, hazard ratio; IM, intermediate metabolizer; KM–LR. Kaplan–Meier curves and Log-Rank test; MITOX, mitoxantrone; OR, odds ratio; OS, overall survival; PFS, progression-free survival; RR, relative risk; TEC, docetaxel, epirubicine, and cyclophosphamide; TTP, time to progression. ^a^Nadir was defined as the lowest count documented within 21 days of doxorubicin administration; ^b^The hypersensitivity reactions were characterized by acute dyspnea, flushing of the face, chest constraint, hypotension, and rash; ^c^χ^2^ test*.

**Table 5:** **Null associations between polymorphisms in xenobiotic metabolizing enzymes and breast cancer outcomes after chemotherapy**.

Gene	SNP	Reference	Population	Protocol	*N*	Outcome	Compared groups
*CBR3*	*730G* > *A*	Fan et al. (2008)	Neoadjuvant	Doxorubicin and docetaxel	99	Hematological toxicity or tumor reduction^a^	*GG, GA*, and AA
*CYP2B6*	*3, *5, *8	Bray et al. (2010)	Adjuvant	AC	230	DFS and toxicity	*1/*1 vs. any variant
	*2, *3, *4, *5, *8, and *9	Gor et al. (2010)	Adjuvant	CAF (+ CTX/thiotepa)	350	DFS	*1/*1 vs. any variant
	*9	Yao et al. (2010)	Adjuvant	CAF or CMF	449	Hematological toxicity	*GG* vs. *GT* or *TT*
*CYP2C8*	*1, *2, *3, and *4	Rizzo et al. (2010)	Adjuvant, neoadjuvant, metastatic	Taxanes	95	Hematological toxicity, neurotoxicity, and hypersensitivity^b^	*1/*1 vs. any variant
*CYP2C9*	*2 and *3	Bray et al. (2010)	Adjuvant	AC	230	BCSS and toxicity	*1/*1 vs. any variant
		Gor et al. (2010)	Adjuvant	CAF (+ CTX/thiotepa)	350	DFS	*1/*1 vs. any variant
*CYP2C19*	*2	Bray et al. (2010)	Adjuvant	AC	230	BCSS and toxicity	*1/*1 vs. any variant
*CYP2D6*	*10	Gor et al. (2010)	Adjuvant	CAF (+ CTX/thiotepa)	350	DFS	*G/G* vs. *G/A* vs. *A/A*
*CYP3A4*	*4	Tsai et al. (2009)	Adjuvant or neoadjuvant	TEC	59	Toxicity	*1/*1 vs. *1/*4
	*5	Tsai et al. (2009)	Adjuvant or neoadjuvant	TEC	59	Toxicity	*1/*1 vs. *1/*5
	*18	Tsai et al. (2009)	Adjuvant or neoadjuvant	TEC	59	Toxicity	*1/*1 vs. *1/*18
	*1B	Yao et al. (2010)	Adjuvant	CAF or CMF	456	Hematological toxicity	*1/*1 vs. *1/*1B or *1B/*1B
	*1G	Zhang et al. (2011)	Neoadjuvant	EPI and CTX	120	Pathological response and toxicity	*CC* vs. *CT* vs. *TT*
*CYP3A5*	*3	Bray et al. (2010)	Adjuvant	AC	230	BCSS and toxicity	*1/*1 vs. any variant
		Gor et al. (2010)	Adjuvant	CAF (+ CTX/thiotepa)	350	DFS	*1/*3, *1/*1, *3/*3
		Zhang et al. (2011)	Neoadjuvant	EPI and CTX	120	Pathological response and toxicity	
	*6	Gor et al. (2010)	Adjuvant	CAF (+ CTX/thiotepa)	350	DFS	*1/*1 vs. *1/*6
*CYP3A7*	*2	Zhang et al. (2011)	Neoadjuvant	EPI and CTX	120	Pathological response and toxicity	*AA, AT, TT*
*GSTA1*	rs3957356-69A > G	Yao et al. (2010)	Adjuvant	CAF or CMF	414	Hematological toxicity	*GG* vs. *GA* or *AA*
*GSTM1*	*Null*	Gor et al. (2010)	Adjuvant	CAF (+ CTX/thiotepa)	350	DFS	Null vs. non-null
		Oliveira et al. (2010)	Neoadjuvant	FEC	40	Pathological response	Null vs. non-null
		Mishra et al. (2011)	Neoadjuvant	CAF – docetaxel	45	Pathological response	Null vs. non-null
		Saadat et al. (2012)	Neoadjuvant	CAF or TAC	101	Pathological response	Null vs. non-null
*GSTO2*	rs156697 (Asn142Asp)	Saadat et al. (2012)	Neoadjuvant	CAF or TAC	101	Pathological response	*Asn/Asn, Asn/Asp, Asp/Asp*
*GSTP1*	*Ala114Val*	Bewick et al. (2008)	Metastatic	MITOX and CTX	95	PFS and BCSS	*AA, AG, GG*
	*Ile105Val*	Bewick et al. (2008)	Metastatic	MITOX and CTX	95	PFS and BCSS	*CC, CT, TT or GG, GA, AA*
		Gor et al. (2010)	Adjuvant	CAF (+ CTX/thiotepa)	350	DFS	*GG, GA, AA*
		Oliveira et al. (2010)	Neoadjuvant	FEC	40	Pathological response	*Ile/Val* vs. *Ile/Ile*
		Yao et al. (2010)	Adjuvant	CAF or CMF	874	DFS	*GG, GA, AA*
*GSTT1*	*Null*	Gor et al. (2010)	Adjuvant	CAF (+ CTX/thiotepa)	350	DFS	Null vs. non-null
		Oliveira et al. (2010)	Neoadjuvant	FEC	40	Pathological response	Null vs. non-null
		Mishra et al. (2011)	Neoadjuvant	CAF – docetaxel	45	Pathological response	Null vs. non-null
		Saadat et al. (2012)	Neoadjuvant	CAF or TAC	101	Pathological response	Null vs. non-null
*GSTZ1*	rs7975 *(Glu32Lys)*	Saadat et al. (2012)	Neoadjuvant	CAF or TAC	101	Pathological response	*Glu/Glu, Glu/Lys, Lys/Lys*

*Abbreviations: AC, adriamycin and cyclophosphamide; BCSS, breast cancer-specific survival; CAF, cyclophosphamide, adriamycin, and 5-fluorouracil; CMF, cyclophosphamide, metothrexate, and 5-fluorouracil; CTX, cyclophosphamide; DFS, disease-free survival; EPI, epirubicine; FEC, 5-fluorouracil, epirubicine, and cyclophosphamide; MITOX, mitoxantrone; OR, odds ratio; OS, overall survival; PFS, progression-free survival; TEC, docetaxel, epirubicine, and cyclophosphamide. ^a^Tumor reduction was defined as ≥25% tumor reduction after the first cycle of doxorubicin; ^b^The hypersensitivity reactions were characterized by acute dyspnea, flushing of the face, chest constraint, hypotension, and rash*.

Among the 20 studies involving tamoxifen use, 16 explored the effects of *CYP2D6* polymorphisms, either considering polymorphisms individually or in their combined genotypes and expected phenotypes. The other four articles evaluated polymorphisms in other genes different from *CYP2D6*. Thus, Nowell et al. (2002) examined the relationship between the SULT1A1*2 allele and survival in a cohort of 337 women (160 receiving tamoxifen); Tucker et al. (2005) evaluated the impact of *CYP3A5* polymorphisms on tamoxifen side effects in 98 postmenopausal women; Jernström et al. (2009) investigated the frequency of *CYP2C8* and *CYP2C9* polymorphisms in relation to disease-free survival in a prospective series of 652 breast cancer patients (297 receiving tamoxifen), and Ruiter et al. (2010) evaluated the impact of CYP2C19*2 and CYP2C19*3 on breast cancer mortality rate in 215 breast cancer patients (80 using tamoxifen). Among the 16 studies evaluating *CYP2D6* polymorphisms, 7 studies also analyzed other genes: Nowell et al. (2005) analyzed *UGT2B15* polymorphisms; Wegman et al. (2005) examined variants of *SULT1A1*; Wegman et al. (2007) evaluated *CYP3A5, SULT1A1*, and *UGT2B15*; Okishiro et al. (2009) analyzed *CYP2C19*; Kiyotani et al. (2010) did not evaluate polymorphisms involving xenobiotic metabolizing enzymes, but examined gene polymorphisms affecting drug transporters (ABCB1, ABCC2, ABCG2); and Moyer et al. (2011) examined *SULT1A1* and *CYP2C19* polymorphisms.

The compiled results in Table 1 indicate that some functional polymorphisms affecting the enzymatic activities of CYP2D6, CYP3A5, CYP2C8, CYP2C19, SULT1A1, or UGT2B15 could significantly affect breast cancer outcomes. Thus, CYP3A5*3 and CYP2C19*2 seem to be beneficial with regards to the risk of recurrence or to breast cancer-specific survival, respectively. All the other polymorphisms with reported significant effects on breast cancer clinical outcomes, i.e., CYP2C8*3, CYP2D6*4, CYP2D6*6, CYP2D6*10, SULT1A1*2, and UGT2B15*2, were associated with an apparent worse response to tamoxifen, resulting in higher risk of tumor recurrence, shorter breast cancer-specific survival, or shorter overall survival.

With regards to CYP2D6*4, the work by Wegman et al. (2007) represents an exception, since it shows an apparent beneficial effect of the genotype CYP2D6*1/*4 in relation to recurrence-free survival. This apparently protective effect, however, was not seen for patients homozygous for the allele *4, who were similar to patients with the wild-type genotype in relation to recurrence-free survival. Although there are no other reports suggesting any beneficial effect of *CYP2D6* polymorphisms in relation to breast cancer outcomes, there is much inconsistency in the supposed detrimental effects of the variant alleles with regards to the risk of recurrence. Thus, only Bijl et al. (2009), with a very limited number of patients, reported an apparent trend between heterozygous and homozygous variants of the allele *4. Goetz et al. (2005) only found significant effects when comparing the homozygous variant genotype *4/*4 to the homozygous wild-type genotype *1/*1. All the other published studies involving CYP2D6*4 indicate no significant effect for the variant allele, either in heterozygosis or in homozygosis (Table 2). The lack of significant independent effect of the allele *4 cannot be attributed to low statistical power, since it was confirmed by the two largest studies (Abraham et al., 2010; Regan et al., 2012). The study by Abraham et al. (2010) also show no significant effects on breast cancer outcomes regarding the *CYP2D6* variant alleles *5, *9, *10, and *41. The lack of significant effects with the variant genotypes of CYP2D6*10 had already been reported by Okishiro et al. (2009) and Toyama et al. (2009).

Other studies analyzed multiple *CYP2D6* polymorphisms (alleles *3, *4, *5, *6, *7, *10, *14, *21, *36–*36, *41) in different combinations, and, based on their expected functional impact on the activity of CYP2D6 enzyme, characterized individual metabolizing phenotypes (Tables 1 and 2). Although some studies have suggested that reduced CYP2D6 enzymatic activity might contribute for higher risk of breast cancer recurrence (Table 1), the recent results of the large studies by Abraham et al. (2010) and Regan et al. (2012) indicate no significant differences in relation to breast cancer-specific survival, either for poor or intermediate CYP2D6 metabolizers, which were characterized based on alleles *3, *4, *5, *6, *7, *10, and *41 (Table 2).

With regards to other polymorphisms that could affect tamoxifen metabolism, there are fewer studies available, and none of them are based on multicenter large populations. In addition, the published data are more diverse in relation to the polymorphisms and to the outcomes that were analyzed, with some polymorphisms being evaluated in only one study. Therefore, the available information does not support combined analyses, and any general conclusion is only tentative.

The only study involving *CYP2C8* (alleles *3 and *4) or *CYP2C9* (alleles *2 and *3) was the work by Jernström et al. (2009), who described an increased risk of early breast cancer-related deaths associated with the haplotype CYP2C8*1/*3/CYP2C9*1/*2 (Table 1). According to the authors, the effect appears to be driven by CYP2C8*3, which presents strong, but not complete linkage disequilibrium with CYP2C9*2. The authors found no significant effects associated with CYP2C8*4, CYP2C9*2, or CYP2C9*3, when evaluated independently (Table 2). With regards to *CYP2C19*, Okishiro et al. (2009) analyzed the alleles *2 and *3, Ruiter et al. (2010) analyzed only the allele *2, and Moyer et al. (2011) analyzed the allele *17. Ruiter et al. (2010) were the only ones to describe an apparent protective effect for the allele *2, with longer breast cancer-specific survival among patients carrying any variant genotype (Table 1). *CYP3A5* was analyzed by Goetz et al. (2005), Tucker et al. (2005), and Wegman et al. (2007). Tucker et al. (2005) evaluated CYP3A5*3 and CYP3A5*6, whereas the other two studies analyzed only CYP3A5*3. Wegman et al. (2007) reported a significantly improved recurrence-free survival among CYP3A5*3-homozygous patients (Table 1), which was not seen by Goetz et al., 2005; Table 2). Tucker et al. (2005) did not evaluate response outcomes, and reported no significant association between *CYP3A5* polymorphisms and adverse events during tamoxifen treatment (Table 2). *SULT1A1* was studied by Nowell et al. (2002), Wegman et al. (2005), Wegman et al. (2007), and Moyer et al. (2011). The first three studies evaluated the polymorphism SULT1A1*2, whereas Moyer et al. (2011) analyzed the number of gene copies in breast tumors. The study by Nowell et al. (2002) was the only one to report a significant effect of the homozygous *2/*2 on patients’ overall survival (Table 1). Wegman et al. (2007) also reported significant detrimental effects of the variant allele *2 on recurrence-free survival after 2 years of tamoxifen treatment (Table 1), but this association was no longer existent after 5 years of tamoxifen treatment (Table 2). The polymorphisms UGT1A8*3, UGT2B7*2, and UGT2B15*2 were analyzed by Ahern et al. (2011), who found no significant associations between their variant alleles and recurrence-free survival in breast cancer patients under adjuvant or neoadjuvant treatment with tamoxifen (Table 2). UGT2B15*2 was also analyzed by Nowell et al. (2002) and Wegman et al. (2007). Nowell et al. (2002) were the only ones to report a significant higher risk of disease progression for patients carrying the variant allele *2 (either in heterozygosis or in homozygosis) in combination with the genotype SULT1A1*2/*2 (Table 1). In conclusion, there is no consistency among the few and sparse results regarding polymorphisms in xenobiotic metabolizing enzymes different from CYP2D6 and their impact on tamoxifen efficacy or safety. Taken together, these results do not suggest clinically relevant implications of such pharmacogenetic targets for tamoxifen treatment.

The published data regarding functional polymorphisms in xenobiotic metabolizing enzymes and breast cancer chemotherapy present even greater diversity in design than observed for studies involving tamoxifen. As expected, these studies involve different patients’ subgroups, and comprise distinct chemotherapeutic protocols. The study designs are also very diverse in the selection of polymorphisms and in analyzed outcomes, with some studies evaluating response outcomes, whereas others focus only in adverse effects. Table 3 summarizes all the available data for significant associations with response outcomes. The compiled information indicates worse outcomes (higher recurrence risk or shorter breast cancer-specific survival) for the polymorphisms CYP1A1m2, CYP1B1*3, CYP2B6*2, *4, and *9, CYP3A4*1B, GSTP1A313G, and *UGT2B7His268Tyr*, whereas a beneficial effect is observed for the variant genotype GSTA1*B/*B (better overall survival) and for the *GSTT1*Null genotype (longer breast cancer-specific survival). Bewick et al. (2008) found no significant effect for *GSTP1A313G* when evaluated independently, but reported a combined effect of *GSTP1A313G* variant genotypes and *SOD2C16CT* variant homozygous genotype resulting in shorter progression-free survival and breast cancer-specific survival for patients using mitoxantrone and cyclophosphamide.

Table 4 summarizes all the available data for significant associations with chemotherapy-related toxicity outcomes. The compiled results indicate higher risk of severe hematological reactions (neutropenia, leucopenia, or thrombocytopenia) for patients with *CBR3 G11A* or *GSTP1A313G* variant genotypes. The other analyzed polymorphisms (CYP1B1*3, CYP2B6*2, CYP3A5*3) showed beneficial effects in relation to the risk of hematological toxicities for patients using anthracycline-based protocols and/or taxanes.

Finally, Table 5 summarizes all the null associations involving polymorphisms in xenobiotic metabolizing enzymes and breast cancer outcomes after chemotherapy. The compiled data comprise various studies evaluating many polymorphisms, but, in most cases, there is only one study for each polymorphism. Because most studies are based on a relatively small number of patients using specific protocols, the compiled information is very diffuse and does not allow definite conclusions or general assumptions. Therefore, the results must be analyzed independently, considering the particularities of each study.

The only polymorphisms that were analyzed by at least two independent studies with similar designs were CYP2B6*2, *3, *5, *8, and *9, CYP2C9 *2 and *3, and CYP3A5*3, which were studied by Bray et al. (2010) and Gor et al. (2010), evaluating survival in patients under adjuvant chemotherapy with anthracycline-based protocols. *GSTM1null* was also studied under similar conditions by Oliveira et al. (2010), Mishra et al. (2011), and Saadat et al. (2012), who evaluated the pathological response to neoadjuvant chemotherapy with anthracyclines and taxanes. In the case of CYP2B6*2, Bray et al. (2010) reported a significant association between the heterozygous variant genotype and shorter time to progression for patients under adjuvant chemotherapy with the protocol AC (Table 3), whereas Gor et al. (2010) found no significant effect on disease-free survival for patients under adjuvant chemotherapy with the protocol CAF (+ CTX/Thiotepa; Table 5). In the case of CYP3A5*3, Tsai et al. (2009) found that patients carrying the heterozygous *1/*3 genotype demonstrated more side effects of fever, pleural effusion, and febrile neutropenia than those with the homozygous *3/*3 genotype (Table 4). Bray et al. (2010), evaluating dose delays in adjuvant chemotherapy with AC, found no significant associations with CYP3A5*3 variant alleles (Table 5), which the authors interpreted as no significant differences in clinically relevant toxicities. Zhang et al. (2011), evaluating severe toxicities (grade 3 or 4) to neoadjuvant chemotherapy with EPI and CTX, also found no significant associations with CYP3A5*3 variant alleles (Table 5). It is not clear whether the discrepancies in the association results reported by different studies involving the same polymorphisms can be attributed to distinct chemotherapeutic protocols, or to other uncontrolled causes of variability.

## Discussion

The field of pharmacogenetics (or pharmacogenomics) has developed with the goal of identifying genetic causes of interindividual differences in pharmacological response, and of using such genetic information to predict one individual’s profile of drug safety and efficacy. In this regard, pharmacogenetic studies are designed to evaluate the correlation between genotypes and phenotypes, and, therefore, provide scientific evidence for the implementation of individualized drug prescriptions, as part of a conduct of personalized medicine. Nevertheless, the characterization of phenotypes may not be easy to accomplish, especially in clinical settings, or when they require invasive procedures. In addition, the actual therapeutic goal is the final clinical outcome, which is, therefore, usually taken as the endpoint of pharmacogenetic studies. One limitation, however, is that clinical outcomes are often the result of complex and overlapping variables, which may have different genetic and non-genetic causes. As a consequence, the strict genotype-phenotype correlation may be compromised, and the results of pharmacogenetic studies may be difficult to interpret. This is exactly the scenario of breast cancer treatment: although there are theoretical bases and practical evidences that genetic influences may indeed affect the pharmacological response, there is great uncertainty about the usefulness of the genetic information to actually predict clinical outcomes and even more on the confidence of using such individual genetic information to modify one’s therapeutic conduct.

The first therapeutic target to drive the attention of pharmacogenetic studies to breast cancer therapy was CYP2D6 in view of its apparent strong genotype-phenotype correlation. Thus, various literature reports indicated that genetic variations in *CYP2D6* affect the availability or the functional activity of the corresponding enzyme (Jin et al., 2005), and that such variations in the enzymatic activity ultimately lead to altered levels of tamoxifen metabolites (Lim et al., 2007, 2011; Kiyotani et al., 2010; de Graan et al., 2011; Irvin et al., 2011; Mürdter et al., 2011). In addition, parallel observations indicated that altered levels of the most active tamoxifen metabolite, endoxifen, result in reduced binding to the estrogen receptor and to lower signaling transduction (Coezy et al., 1982; Lim et al., 2006). Taken together, these results have reinforced the notion that *CYP2D6* polymorphisms could be useful to predict one individual’s response to tamoxifen, and that *CYP2D6* genotyping might help the selection of the antiestrogenic drug or the definition of tamoxifen dosing. The promises of this rationale in relation to possible improvements in breast cancer outcomes has led to the expansion of pharmacogenomic studies to other therapeutic targets in breast cancer antineoplastic chemotherapy, as well as to other targets of tamoxifen pharmacokinetics.

The evaluation of the literature production involving the pharmacogenetics of breast cancer indicates a great number of studies. The current review, which was focused on pharmacokinetic targets, and more specifically, on functional polymorphisms of xenobiotic metabolizing enzymes, has initially retrieved 158 references on the subject. Although the criteria for the systematic search included the mention to xenobiotic metabolizing enzymes in the article’s title or abstract, most of the retrieved documents explored different targets as their main research subject. The most frequent targets besides xenobiotic metabolizing enzymes that were identified in the current search were drug transporters, which might contribute to antineoplastics’ pharmacokinetics, affecting drug distribution and disposition, and aromatases, which modulate the availability of estrogens, and the estrogen receptor. The selection of documents exploring functional polymorphisms of xenobiotic metabolizing enzymes resulted in 43 original articles, 23 devoted to tamoxifen, and 20 dealing with various antineoplastic protocols. Among the articles involving tamoxifen, *CYP2D6* was the main pharmacogenetic target, whereas multiple xenobiotic metabolizing enzymes were explored with regards to different cytotoxic antineoplastics. This conjunct of original articles on the pharmacogenetics of breast cancer is still relatively limited in number and very diverse in design. Consequently, general conclusions are difficult to extract at this point, and no clear recommendations can be made for application in clinical practice. Nevertheless, the use of the same terms for bibliographic search without the restriction to original articles indicates that 24 reviews were published on the subject in the same period of time. Although the analysis of the reviews was not part of our systematic search, an overview of the publications indicates that *CYP2D6* predominates as the chosen subject, probably as a reflex of the great enthusiasm after the earliest studies. Some of the most recent reviews already include data on other xenobiotic metabolizing enzymes, but no systematic review had been presented before.

The studies on *CYP2D6* polymorphisms and their influences on tamoxifen efficacy show many discrepancies, which can be accounted to multiple factors, including variations in study design, in the definitions of breast cancer disease, and in the population characteristics. Thus, in relation to study design, different *CYP2D6* polymorphisms are analyzed, and some studies use tumor tissue for genotype assessment, which can compromise the accurate characterization of the number and types of *CYP2D6* alleles. There are also variations in the therapeutic regimen, including adjuvant, neoadjuvant and palliative treatment, and evaluation of different disease outcomes. The prescribed tamoxifen dose may also vary, and, in most studies, there is no control or documentation of drug adherence and of concomitant use of CYP2D6 inhibitors. The population characteristics also present great variability, including different ethnic and cultural backgrounds, which can interfere with risk estimates. Finally, the percentage of postmenopausal women may also be an interfering factor, if there is no control of the use of aromatase inhibitors after the completion of tamoxifen treatment.

A meta-analysis published by Seruga and Amir (2010) analyzed data from 10 studies assessing CYP2D6 genotype and clinical outcomes in breast cancer. The authors found significant heterogeneity in the definition of comparison groups between studies, but suggested that normal CYP2D6 function was associated with a trend toward improved disease-free survival (HR 2.07, 95% CI 0.96–4.49, *P* = 0.06). The most recent work on the subject, by Regan et al. (2012), enrolled a large number of postmenopausal patients treated with tamoxifen for 5 years, as part of the BIG 1–98 trial, and showed no association between CYP2D6 metabolizing phenotype and the risk of recurrence (characterized by the breast cancer-free interval), with or without previous chemotherapy. The patients who were identified as poor or intermediate metabolizers (based on alleles *2, *3, *4, *6, *10, *17, and *41) did not have worse disease outcomes than extensive metabolizers, or higher chance of presenting hot flushes as side effects. The authors argued that CYP2D6 metabolizing phenotype is not the correct surrogate for predicting symptoms or outcome of tamoxifen-treated postmenopausal women, and advocated that *CYP2D6* pharmacogenetic testing to determine whether adjuvant tamoxifen should be given to postmenopausal women with endocrine-responsive breast cancer is not justified (Regan et al., 2012). Although very large in the number of patients enrolled, and with a well-planned prospective design, one limitation of the work by Regan et al. (2012), as acknowledged by the authors, is the use of tumor samples for genotyping, which could lead to some misclassification of metabolizing phenotypes. In addition, the trial did not provide data on concomitant medications, and therefore, a possible interference of CYP2D6 inhibitors cannot be ruled out.

Two other large studies involving breast cancer survivors who used tamoxifen (Abraham et al., 2010; Madlensky et al., 2011) also suggested the lack of association between *CYP2D6* genotypes, or estimated CYP2D6 metabolizing phenotypes, and breast cancer outcomes. Thus, Abraham et al. (2010) found that only the allele *6 was associated with lower breast cancer-specific survival, whereas the other alleles (*4, *5, *9, *10, and *41) had no significant effects. Madlensky et al. (2011) found no significant associations between CYP2D6 metabolizing phenotypes and the risk of breast cancer recurrence or second breast cancer. The latter authors, however, reported that patients with endoxifen concentrations lower than 5.97 ng/mL were at higher risk of breast cancer recurrence, and that the proportion of decreased CYP2D6 metabolizing phenotype was higher in this group. In addition to CYP2D6 metabolizing phenotype, the authors identified other variables, such as excess weight and low tamoxifen levels (suggesting failures in adherence), which were associated with low endoxifen levels, but not independently associated with breast cancer outcomes. The authors suggested the existence of a non-linear dose-response effect for tamoxifen, and proposed that a threshold of endoxifen must be achieved for the therapeutic effect of tamoxifen. Finally, the authors argued that the metabolizing phenotype alone is not enough to determine whether tamoxifen is of potential benefit to any individual patient (Madlensky et al., 2011).

Another aspect with pharmacogenetic interest in relation to tamoxifen is the occurrence of side effects, especially hot flushes, which are intrinsically correlated with the suppression of estrogen signaling, and thus expected to be associated with endoxifen levels. Some authors have proposed that hot flushes could serve as surrogates of tamoxifen efficacy, and that their absence could indicate patients at higher risk of recurrence (Cuzick et al., 2008; Mortimer et al., 2008). As a logical consequence, it has been hypothesized that poor CYP2D6 metabolizers would be less likely to experience moderate to severe hot flushes (Goetz et al., 2005; Henry et al., 2009), whereas, in contrast, extensive metabolizers would be more prone to prematurely discontinue tamoxifen, possibly as a consequence of severe hot flushes (Rae et al., 2009). The assumed correlation between CYP2D6 metabolizing phenotypes and hot flushes, however, was based on few and weak associations, with no (or only border-line) statistical significance, which were not validated in the recent large prospective study by Regan et al. (2012). Unfortunately, there have been no similar large observational studies evaluating CYP2D6 metabolizing phenotypes and hot flushes as possible causes of non-adherence or non-persistency to tamoxifen treatment. It would be also interesting to have studies evaluating the correlation between endoxifen levels and the occurrence of severe hot flushes.

The metabolism of tamoxifen involves other metabolizing enzymes than CYP2D6, such as CYP3A4/5, 2C8/9, SULT1A1, UGT1A8, UGT2B7, and UGT2B15, which might also have an impact on the availability of endoxifen and other metabolites, and therefore contribute for the heterogeneity in breast cancer outcomes. However, there are few studies evaluating genetic polymorphisms on such metabolic activities and their consequences on tamoxifen efficacy. In addition, the assumption that tamoxifen efficacy is mainly dependent on the availability of endoxifen must also be considered with a certain caution. Although endoxifen has greater affinity for the estrogen receptor than tamoxifen or *N*-desmethyl-tamoxifen (Coezy et al., 1982; Jordan, 1982; Robertson et al., 1982), and higher plasma concentrations than 4-hydroxy-tamoxifen (Johnson et al., 2004; Lim et al., 2005), it has been estimated that tamoxifen and its metabolites other than endoxifen are capable of nearly saturating estrogen receptors, with 99.94% occupancy (Dowsett and Haynes, 2003). Therefore, impaired tamoxifen metabolism may not represent a full limitation for tamoxifen efficacy, other cellular mechanisms must be considered when evaluating tamoxifen resistance. Thus, Kim et al. (2011) have shown that tumor cells with low mRNA expression of the estrogen receptor (*ESR1*) present increased tamoxifen resistance when compared to cells with high-level mRNA expression, regardless of endoxifen concentrations. Finally, recent studies have suggested that tamoxifen and its metabolites may have secondary pharmacological actions, such as blockade of voltage-dependent Ca^2+^ channels (Kuo et al., 2012), vasodilation (Montenegro et al., 2011), and aromatase inhibition (Lu et al., 2012). The possible impact of such additional pharmacological mechanisms on breast cancer is not known yet.

The evaluation of studies involving chemotherapeutic protocols for breast cancer therapy indicates that many xenobiotic metabolizing enzymes other than CYP2D6 are also being considered as possible pharmacogenetic targets. These studies indicate great diversity of antineoplastic protocols and a balanced interest in both efficacy and toxicity. The number of studies, however, is still very limited, with only one or very few studies for each pharmacogenetic target, and no confirmation of positive associations. The only exception appears to involve *GSTP1 Ile105Val*, since three different studies (Zárate et al., 2007; Yao et al., 2010; Zhang et al., 2011), exploring anthracycline-based protocols, suggest higher risk of severe hematological toxicity (neutropenia or leucopenia) for patients with variant genotypes. Although there are some inconsistencies regarding the heterozygous genotype, the combined results appear to indicate that the presence of valine instead of isoleucine, which results in decreased enzymatic activity (Watson et al., 1998), would favor higher plasma concentrations of the chemotherapeutic drugs, with consequent increased toxicity. Such increased toxicity, as an apparent consequence of increased plasma concentrations, does not seem to have a direct correlation with better response profile. Thus, although Zhang et al. (2011) have reported better pathological response for patients with the *G/G (Val/Val)* genotype after neoadjuvant therapy, Huang et al. (2008) found that patients with variant genotypes had higher rates of early relapse after adjuvant treatment, and other authors found no significant effects of the *GSTP1 Ile105Val* polymorphism on the therapeutic response after adjuvant (Gor et al., 2010; Yao et al., 2010) or neoadjuvant treatment (Oliveira et al., 2010).

The above results point an important aspect of pharmacogenetic studies in clinical oncology, which is the apparent higher variability in the results involving response outcomes than in those related to drugs’ toxicities. This is not surprising considering that, in addition to the subjects’ polymorphisms, tumors might also present mutations, as well as epigenetic variations that could affect cellular response to chemotherapy. Secondly, an individual clinical response also involves many non-genetic factors that are difficult to control, such as lifestyle, comorbidities, drug interactions, and treatment adherence. Finally, in the case of breast cancer, clinical outcomes have a long time frame, which make them more difficult to study in a controlled design. Although it is logical that prospective observational studies combining the evaluation of pharmacogenetic data and non-genetic patients’ characteristics would be ideal for modeling breast cancer clinical outcomes, their practical implementation is certainly a challenge.

Another important issue raised by the example of the results involving *GSTP1 Ile105Val* involves the translation of pharmacogenetic data into the clinical practice of oncology. Let’s assume that a genotype-phenotype is established, and that it involves a pharmacokinetic target, with an apparent plasma concentration-dependent relationship. In such scenario, should one individual’s dose be adjusted based on the identified genotype? For example, should patients with the homozygous variant *GSTP1* genotype receive lower doses as an attempt to reduce their risk of severe neutropenia, and therefore avoid treatment delays or interruption that could compromise the final response outcomes? Although the genotype-phenotype relationship might be recognized when evaluating the risk estimates in a population, there is no absolute correspondence between genotypes and outcomes at the individual level. In addition, there is certainly great concern about reducing the dose, and consequently increasing the risk of lower response. Thus, it seems unlikely that such prophylactic dose adjustments based on an individual genotype would even be tested in a clinical trial, and ultimately incorporated for oncological treatments. Alternatively, should patients at estimated higher risk of toxicity receive some prophylactic or supportive treatment? For example, in the case of patients with the variant *GSTP1* genotype, should stimulating factors be considered as a strategy to minimize their risk of neutropenia? There is no doubt that the idea of being able to choose a drug guided by an individual genotype, as it was initially considered for tamoxifen vs. aromatase inhibitors, is more appealing as a practical pharmacogenetic conduct in oncology than the hard and risky task of defining one individual’s antineoplastic dose. Nevertheless, the current recommendations of the American Society of Clinical Oncology (ASCO) do not include routine *CYP2D6* testing to select the endocrine therapy (Burstein et al., 2010).

## Conclusion and Perspectives

The application of pharmacogenetics to predict breast cancer therapeutic outcomes and to select one individual’s chemotherapeutic protocol is still far from clinical routine. Most studies used the candidate-gene approach, and evaluated single or few SNPs in metabolic pathways. In addition, because of the difficulties of conducting large trials, most studies explored the most common genetic variations. Although some very interesting results have been produced, no clear practical recommendations are recognized yet. The current challenge is to simultaneously evaluate multiple genes and pathways, including rarer variants, and to consider their combined effects on drug efficacy and toxicity. Such endeavor will require large, multicentric studies, and longer and well-controlled follow-ups, in order to produce reliable information, aiming at consequent practical applications for breast cancer therapy.

These above conclusions regarding the constraints for clinical applicability of pharmacogenomic data in breast cancer management meet the consensus view on the use of qualifying biomarkers in drug safety (Agúndez et al., 2012). It appears, thus, that the limitations of pharmacogenomic studies are not a particularity of Oncology, and that the use of genetic information as biomarkers requires medical and scientific consensus and the development of adequate guidelines for clinical practice. Research consortia appear to be good opportunities to explore these goals.

## Authors’ Contributions

Juliana Simões Festa-Vasconcellos performed the bibliographic searches, retrieved and selected abstracts, draw the Figure, helped reviewing the data from tables, and helped revising the manuscript. Sheyla Maria Torres Goulart-Citrangulo reviewed the articles involving tamoxifen, and compiled the data in Tables 1 and 2. Marcelo Sobral Leite reviewed the articles involving any kind of chemotherapy, and compiled the data in Tables 3– 5. Rosane Vianna-Jorge conceived, designed, and coordinated the study, reviewed, and analyzed the data, wrote, and revised the manuscript. All authors read and approved the final manuscript.

## Conflict of Interest Statement

The authors declare that the research was conducted in the absence of any commercial or financial relationships that could be construed as a potential conflict of interest.

## References

[B1] Abraham J. E. Maranian M. J. Driver K. E. Platte R. Kalmyrzaev B. Baynes C. (2010). CYP2D6 gene variants: association with breast cancer specific survival in a cohort of breast cancer patients from the United Kingdom treated with adjuvant tamoxifen. Breast Cancer Res. 12, R64 10.1186/bcr2717 20731819 PMC2949659

[B2] Agúndez J. A. Abad-Santos F. Aldea A. Alonso-Navarro H. Bernal M. L. Borobia A. M. (2012). Toward a clinical practice guide in pharmacogenomics testing for functional polymorphisms of drug-metabolizing enzymes. Gene/drug pairs and barriers perceived in Spain. Front. Genet. 3:273 10.3389/fgene.2012.00273 23233861 PMC3516180

[B3] Ahern T. P. Christensen M. Cronin-Fenton D. P. Lunetta K. L. Søiland H. Gjerde J. (2011). Functional polymorphisms in UDP-glucuronosyl transferases and recurrence in tamoxifen-treated breast cancer survivors. Cancer Epidemiol. Biomarkers Prev. 20, 1937–1943 10.1158/1055-9965.EPI-11-0419 21750172 PMC3169710

[B4] Anderson W. F. Matsuno R. (2006). Breast cancer heterogeneity: a mixture of at least two main types? J. Natl. Cancer Inst. 98, 948–951 10.1093/jnci/djj295 16849671

[B5] Banerji S. Cibulskis K. Rangel-Escareno C. Brown K. K. Carter S. L. Frederick A. M. (2012). Sequence analysis of mutations and translocations across breast cancer subtypes. Nature 486, 405–409 10.1038/nature11154 22722202 PMC4148686

[B6] Berry D. A. Cronin K. A. Plevritis S. K. Fryback D. G. Clarke L. Zelen M. (2005). Effect of screening and adjuvant therapy on mortality from breast cancer. N. Engl. J. Med. 353, 1784–1792 10.1056/NEJMoa050518 16251534

[B7] Bewick M. A. Conlon M. S. C. Lafrenie R. M. (2008). Polymorphisms in manganese superoxide dismutase, myeloperoxidase and glutathione-S-transferase and survival after treatment for metastatic breast cancer. Breast Cancer Res. Treat. 111, 93–101 10.1007/s10549-007-9764-8 17922231

[B8] Bijl M. J. van Schaik R. H. N. Lammers L. A. Hofman A. Vulto A. G. van Gelder T. (2009). The CYP2D6*4 polymorphism affects breast cancer survival in tamoxifen users. Breast Cancer Res. Treat. 118, 125–130 10.1007/s10549-008-0272-2 19189212

[B9] Bray J. Sludden J. Griffin M. J. Cole M. Verrill M. Jamieson D. (2010). Influence of pharmacogenetics on response and toxicity in breast cancer patients treated with doxorubicin and cyclophosphamide. Br. J. Cancer 102, 1003–1009 10.1038/sj.bjc.6605587 20179710 PMC2844036

[B10] Burstein H. J. Prestrud A. A. Seidenfeld J. Anderson H. Buchholz T. A. Davidson N. E. (2010). American Society of Clinical Oncology clinical practice guideline: update on adjuvant endocrine therapy for women with hormone receptor-positive breast cancer. J. Clin. Oncol. 28, 3784–3796 10.1200/JCO.2009.25.8707 20625130 PMC5569672

[B11] Buzdar A. U. (2001). Endocrine therapy in the treatment of metastatic breast cancer. Semin. Oncol. 28, 291–304 10.1016/S0093-7754(01)90122-8 11402439

[B12] Chacko P. Joseph T. Mathew B. S. Rajan B. Pillai M. R. (2005). Role of xenobiotic metabolizing gene polymorphisms in breast cancer susceptibility and treatment outcome. Mutat. Res. 581, 153–163 10.1016/j.mrgentox.2004.11.018 15725614

[B13] Coezy E. Borgna J. L. Rochefort H. (1982). Tamoxifen and metabolites in MCF7 cells: correlation between binding to estrogen receptor and inhibition of cell growth. Cancer Res. 42, 317–323 7053859

[B14] Cuzick J. Sestak I. Cella D. Fallowfield L. (2008). Treatment-emergent endocrine symptoms and the risk of breast cancer recurrence: a retrospective analysis of the ATAC trial. Lancet Oncol. 9, 1143–1148 10.1016/S1470-2045(08)70259-6 18976959

[B15] Danova M. Delfanti S. Manzoni M. Mariucci S. (2011). Tissue and soluble biomarkers in breast cancer and their applications: ready to use? J. Natl. Cancer Inst. 2011, 75–78 10.1093/jncimonographs/lgr023 22043046

[B16] de Graan A. J. M. Teunissen S. F. de Vos F. Y. F. L. Loos W. J. van Schaik R. H. N. de Jongh F. E. (2011). Dextromethorphan as a phenotyping test to predict endoxifen exposure in patients on tamoxifen treatment. J. Clin. Oncol. 29, 3240–3246 10.1200/JCO.2010.32.9839 21768449

[B17] De Laurentiis M. Cancello G. D’Agostino D. Giuliano M. Giordano A. Montagna E. (2008). Taxane-based combinations as adjuvant chemotherapy of early breast cancer: a meta-analysis of randomized trials. J. Clin. Oncol. 26, 44–53 10.1200/JCO.2008.16.4491 18165639

[B18] DeSantis C. Siegel R. Bandi P. Jemal A. (2011). Breast cancer statistics, 2011. CA Cancer J. Clin. 61, 409–418 10.3322/caac.20134 21969133

[B19] Desta Z. Ward B. A. Soukhova N. V. Flockhart D. A. (2004). Comprehensive evaluation of tamoxifen sequential biotransformation by the human cytochrome P450 system in vitro: prominent roles for CYP3A and CYP2D6. J. Pharmacol. Exp. Ther. 310, 1062–1075 10.1124/jpet.104.065607 15159443

[B20] Dowsett M. Haynes B. P. (2003). Hormonal effects of aromatase inhibitors: focus on premenopausal effects and interaction with tamoxifen. J. Steroid Biochem. Mol. Biol. 86, 255–263 10.1016/S0960-0760(03)00365-0 14623519

[B21] Doyle J. J. Neugut A. I. Jacobson J. S. Grann V. R. Hershman D. L. (2005). Chemotherapy and cardiotoxicity in older breast cancer patients: a population-based study. J. Clin. Oncol. 23, 8597–8605 10.1200/JCO.2005.02.5841 16314622

[B22] Early Breast Cancer Trialists Collaborative Group (EBCTCG) (2005). Effects of chemotherapy and hormonal therapy for early breast cancer on recurrence and 15-year survival: an overview. Lancet 365, 1687–1717 10.1016/S0140-6736(05)66544-0 15894097

[B23] Engels F. K. Loos W. J. van der Bol J. M. de Bruijn P. Mathijssen R. H. J. Verweij J. (2011). Therapeutic drug monitoring for the individualization of docetaxel dosing: a randomized pharmacokinetic study. Clin. Cancer Res. 17, 353–362 10.1158/1078-0432.CCR-10-1636 21224369

[B24] Fan L. Goh B. C. Wong C. I. Sukri N. Lim S. E. Tan S. H. (2008). Genotype of human carbonyl reductase CBR3 correlates with doxorubicin disposition and toxicity. Pharmacogenet. Genomics 18, 621–631 10.1097/FPC.0b013e328301a869 18551042

[B25] Ferlay J. Shin H.-R. Bray F. Forman D. Mathers C. Parkin D. M. (2010). Estimates of worldwide burden of cancer in 2008: GLOBOCAN 2008. Int. J. Cancer 127, 2893–2917 10.1002/ijc.25516 21351269

[B26] Fisher B. Costantino J. P. Wickerham D. L. Redmond C. K. Kavanah M. Cronin W. M. (1998). Tamoxifen for prevention of breast cancer: report of the National Surgical Adjuvant Breast and Bowel Project P-1 Study. J. Natl. Cancer Inst. 90, 1371–1388 10.1093/jnci/90.18.1371 9747868

[B27] Gianni L. Herman E. H. Lipshultz S. E. Minotti G. Sarvazyan N. Sawyer D. B. (2008). Anthracycline cardiotoxicity: from bench to bedside. J. Clin. Oncol. 26, 3777–3784 10.1200/JCO.2007.15.7404 18669466 PMC3018290

[B28] Goetz M. P. Rae J. M. Suman V. J. Safgren S. L. Ames M. M. Visscher D. W. (2005). Pharmacogenetics of tamoxifen biotransformation is associated with clinical outcomes of efficacy and hot flushes. J. Clin. Oncol. 23, 9312–9318 10.1200/JCO.2005.09.119 16361630

[B29] Gor P. P. Su H. I. Gray R. J. Gimotty P. A. Horn M. Aplenc R. (2010). Cyclophosphamide-metabolizing enzyme polymorphisms and survival outcomes after adjuvant chemotherapy for node-positive breast cancer: a retrospective cohort study. Breast Cancer Res. 12, R26 10.1186/bcr2679 20459744 PMC2917014

[B30] Hassan M. S. U. Ansari J. Spooner D. Hussain S. A. (2010). Chemotherapy for breast cancer (review). Oncol. Rep. 24, 1121–1231 10.3892/or_00000963 20878101

[B31] Hayes D. F. Isaacs C. Stearns V. (2001). Prognostic factors in breast cancer: current and new predictors of metastasis. J. Mammary Gland Biol. Neoplasia 6, 375–392 10.1023/A:1014778713034 12013528

[B32] Henry N. L. Rae J. M. Li L. Azzouz F. Skaar T. C. Desta Z. (2009). Association between CYP2D6 genotype and tamoxifen-induced hot flashes in a prospective cohort. Breast Cancer Res. Treat. 117, 571–575 10.1007/s10549-009-0309-1 19153830 PMC2746261

[B33] Hershman D. Neugut A. I. Jacobson J. S. Wang J. Tsai W.-Y. McBride R. (2007). Acute myeloid leukemia or myelodysplastic syndrome following use of granulocyte colony-stimulating factors during breast cancer adjuvant chemotherapy. J. Natl. Cancer Inst. 99, 196–205 10.1093/jnci/djm016 17284714

[B34] Hoskins J. M. Carey L. A. McLeod H. L. (2009). CYP2D6 and tamoxifen: DNA matters in breast cancer. Nat. Rev. Cancer 9, 576–586 10.1038/nrc2683 19629072

[B35] Huang M. Y. Wang Y. H. Chen F. M. Lee S. C. Fang W. Y. Cheng T. L. (2008). Multiple genetic polymorphisms of GSTP1 313AG, MDR1 3435CC, and MTHFR 677CC highly correlated with early relapse of breast cancer patients in Taiwan. Ann. Surg. Oncol. 15, 872–880 10.1245/s10434-008-9968-0 18095031

[B36] Innocenti F. Iyer L. Ramírez J. Green M. D. Ratain M. J. (2001). Epirubicin glucuronidation is catalyzed by human UDP-glucuronosyltransferase 2B7. Drug Metab. Dispos. 29, 686–692 11302935

[B37] Irvin W. J. Walko C. M. Weck K. E. Ibrahim J. G. Chiu W. K. Dees E. C. (2011). Genotype-guided tamoxifen dosing increases active metabolite exposure in women with reduced CYP2D6 metabolism: a multicenter study. J. Clin. Oncol. 29, 3232–3239 10.1200/JCO.2010.31.4427 21768473 PMC3158597

[B38] Jacquin J. P. Jones S. Magné N. Chapelle C. Ellis P. Janni W. (2012). Docetaxel-containing adjuvant chemotherapy in patients with early stage breast cancer. Consistency of effect independent of nodal and biomarker status: a meta-analysis of 14 randomized clinical trials. Breast Cancer Res. Treat. 134, 903–913 10.1007/s10549-011-1933-0 22270929

[B39] Jaiyesimi I. A. Buzdar A. U. Decker D. A. Hortobagyi G. N. (1995). Use of tamoxifen for breast cancer: twenty-eight years later. J. Clin. Oncol. 13, 513–529 7844613 10.1200/JCO.1995.13.2.513

[B40] Jemal A. Bray F. Center M. M. Ferlay J. Ward E. Forman D. (2011). Global cancer statistics. CA Cancer J. Clin. 61, 69–90 10.3322/caac.20107 21296855

[B41] Jernström H. Bågeman E. Rose C. Jönsson P. E. Ingvar C. (2009). CYP2C8 and CYP2C9 polymorphisms in relation to tumour characteristics and early breast cancer related events among 652 breast cancer patients. Br. J. Cancer 101, 1817–1823 10.1038/sj.bjc.6605428 19935798 PMC2788256

[B42] Jin Y. Desta Z. Stearns V. Ward B. Ho H. Lee K.-H. (2005). CYP2D6 genotype, antidepressant use, and tamoxifen metabolism during adjuvant breast cancer treatment. J. Natl. Cancer Inst. 97, 30–39 10.1093/jnci/dji005 15632378

[B43] Johnson M. D. Zuo H. Lee K.-H. Trebley J. P. Rae J. M. Weatherman R. V. (2004). Pharmacological characterization of 4-hydroxy-N-desmethyl tamoxifen, a novel active metabolite of tamoxifen. Breast Cancer Res. Treat. 85, 151–159 10.1023/B:BREA.0000025406.31193.e8 15111773

[B44] Jordan V. C. (1982). Metabolites of tamoxifen in animals and man: identification, pharmacology, and significance. Breast Cancer Res. Treat. 2, 123–138 10.1007/BF01806449 6184101

[B45] Kim C. Tang G. Pogue-Geile K. L. Costantino J. P. Baehner F. L. Baker J. (2011). Estrogen receptor (ESR1) mRNA expression and benefit from tamoxifen in the treatment and prevention of estrogen receptor-positive breast cancer. J. Clin. Oncol. 29, 4160–4167 10.1200/JCO.2010.32.9615 21947828 PMC3208536

[B46] Kiyotani K. Mushiroda T. Imamura C. K. Hosono N. Tsunoda T. Kubo M. (2010). Significant effect of polymorphisms in CYP2D6 and ABCC2 on clinical outcomes of adjuvant tamoxifen therapy for breast cancer patients. J. Clin. Oncol. 28, 1287–1293 10.1200/JCO.2009.25.7246 20124171 PMC4872305

[B47] Kuo J.-R. Wang C.-C. Huang S.-K. Wang S.-J. (2012). Tamoxifen depresses glutamate release through inhibition of voltage-dependent Ca2+ entry and protein kinase Cα in rat cerebral cortex nerve terminals. Neurochem. Int. 60, 105–114 10.1016/j.neuint.2011.11.014 22142530

[B48] Lal S. Mahajan A. Chen W. N. Chowbay B. (2010). Pharmacogenetics of target genes across doxorubicin disposition pathway: a review. Curr. Drug Metab. 11, 115–128 10.2174/138920010791110890 20302569

[B49] Lim H. S. Ju Lee H. Seok Lee K. Sook Lee E. Jang I. J. Ro J. (2007). Clinical implications of CYP2D6 genotypes predictive of tamoxifen pharmacokinetics in metastatic breast cancer. J. Clin. Oncol. 25, 3837–3845 10.1200/JCO.2007.11.4850 17761971

[B50] Lim J. S. L. Chen X. A. Singh O. Yap Y. S. Ng R. C. H. Wong N. S. (2011). Impact of CYP2D6, CYP3A5, CYP2C9 and CYP2C19 polymorphisms on tamoxifen pharmacokinetics in Asian breast cancer patients. Br. J. Clin. Pharmacol. 71, 737–750 10.1111/j.1365-2125.2011.03905.x 21480951 PMC3093079

[B51] Lim Y. C. Desta Z. Flockhart D. A. Skaar T. C. (2005). Endoxifen (4-hydroxy-N-desmethyl-tamoxifen) has anti-estrogenic effects in breast cancer cells with potency similar to 4-hydroxy-tamoxifen. Cancer Chemother. Pharmacol. 55, 471–478 10.1007/s00280-004-0926-7 15685451

[B52] Lim Y. C. Li L. Desta Z. Zhao Q. Rae J. M. Flockhart D. A. (2006). Endoxifen, a secondary metabolite of tamoxifen, and 4-OH-tamoxifen induce similar changes in global gene expression patterns in MCF-7 breast cancer cells. J. Pharmacol. Exp. Ther. 318, 503–512 10.1124/jpet.105.100511 16690721

[B53] Lu W. J. Desta Z. Flockhart D. A. (2012). Tamoxifen metabolites as active inhibitors of aromatase in the treatment of breast cancer. Breast Cancer Res. Treat. 131, 473–481 10.1007/s10549-011-1428-z 21390495

[B54] Madlensky L. Natarajan L. Tchu S. Pu M. Mortimer J. Flatt S. W. (2011). Tamoxifen metabolite concentrations, CYP2D6 genotype, and breast cancer outcomes. Clin. Pharmacol. Ther. 89, 718–725 10.1038/clpt.2011.32 21430657 PMC3081375

[B55] Marsh S. Somlo G. Li X. Frankel P. King C. R. Shannon W. D. (2007). Pharmacogenetic analysis of paclitaxel transport and metabolism genes in breast cancer. Pharmacogenomics J. 7, 362–365 10.1038/sj.tpj.6500434 17224914

[B56] Martín M. Seguí M. A. Antón A. Ruiz A. Ramos M. Adrover E. (2010). Adjuvant docetaxel for high-risk, node-negative breast cancer. N. Engl. J. Med. 363, 2200–2210 10.1056/NEJMoa0910320 21121833

[B57] Mishra A. Chandra R. Mehrotra P. K. Bajpai P. Agrawal D. (2011). Glutathione S-transferase M1 and T1 polymorphism and response to neoadjuvant chemotherapy (CAF) in breast cancer patients. Surg. Today 41, 471–476 10.1007/s00595-009-4310-4 21431478

[B58] Montenegro M. F. Ceron C. S. Salgado M. C. O. Desta Z. Flockhart D. A. Tanus-Santos J. E. (2011). Tamoxifen and its metabolites cause acute vasorelaxation of aortic rings by inducing vasodilator prostanoid synthesis. J. Cardiovasc. Pharmacol. 58, 647–653 10.1097/FJC.0b013e31823171ba 21885992

[B59] Mortimer J. E. Flatt S. W. Parker B. A. Gold E. B. Wasserman L. Natarajan L. (2008). Tamoxifen, hot flashes and recurrence in breast cancer. Breast Cancer Res. Treat. 108, 421–426 10.1007/s10549-007-9612-x 17541741 PMC2575100

[B60] Moyer A. M. Suman V. J. Weinshilboum R. M. Avula R. Black J. L. Safgren S. L. (2011). SULT1A1, CYP2C19 and disease-free survival in early breast cancer patients receiving tamoxifen. Pharmacogenomics 12, 1535–1543 10.2217/pgs.11.97 21961651 PMC3235041

[B61] Mürdter T. E. Schroth W. Bacchus-Gerybadze L. Winter S. Heinkele G. Simon W. (2011). Activity levels of tamoxifen metabolites at the estrogen receptor and the impact of genetic polymorphisms of phase I and II enzymes on their concentration levels in plasma. Clin. Pharmacol. Ther. 89, 708–717 10.1038/clpt.2011.27 21451508

[B62] Newman W. G. Hadfield K. D. Latif A. Roberts S. A. Shenton A. McHague C. (2008). Impaired tamoxifen metabolism reduces survival in familial breast cancer patients. Clin. Cancer Res. 14, 5913–5918 10.1158/1078-0432.CCR-07-5235 18794105

[B63] Nik-Zainal S. Alexandrov L. B. Wedge D. C. Van Loo P. Greenman C. D. Raine K. (2012). Mutational processes molding the genomes of 21 breast cancers. Cell 149, 979–993 10.1016/j.cell.2012.04.024 22608084 PMC3414841

[B64] Nowell S. Sweeney C. Winters M. Stone A. Lang N. P. Hutchins L. F. (2002). Association between sulfotransferase 1A1 genotype and survival of breast cancer patients receiving tamoxifen therapy. J. Natl. Cancer Inst. 94, 1635–1640 10.1093/jnci/94.21.1635 12419790

[B65] Nowell S. A. Ahn J. Rae J. M. Scheys J. O. Trovato A. Sweeney C. (2005). Association of genetic variation in tamoxifen-metabolizing enzymes with overall survival and recurrence of disease in breast cancer patients. Breast Cancer Res. Treat. 91, 249–258 10.1007/s10549-004-7751-x 15952058

[B66] O’Donnell P. H. Ratain M. J. (2012). Germline pharmacogenomics in oncology: decoding the patient for targeting therapy. Mol. Oncol. 6, 251–259 10.1016/j.molonc.2012.01.005 22321460 PMC5528362

[B67] Okishiro M. Taguchi T. Jin Kim S. Shimazu K. Tamaki Y. Noguchi S. (2009). Genetic polymorphisms of CYP2D6 10 and CYP2C19 2, 3 are not associated with prognosis, endometrial thickness, or bone mineral density in Japanese breast cancer patients treated with adjuvant tamoxifen. Cancer 115, 952–961 10.1002/cncr.24111 19156902

[B68] Oliveira A. L. Rodrigues F. F. O. Santos R. E. Aoki T. Rocha M. N. Longui C. A. (2010). GSTT1, GSTM1, and GSTP1 polymorphisms and chemotherapy response in locally advanced breast cancer. Genet. Mol. Res. 9, 1045–1053 10.4238/vol9-2gmr726 20568049

[B69] Osborne C. K. (1998). Tamoxifen in the treatment of breast cancer. N. Engl. J. Med. 339, 1609–1618 10.1056/NEJM199811263392207 9828250

[B70] Parmar S. Stingl J. C. Huber-Wechselberger A. Kainz A. Renner W. Langsenlehner U. (2011). Impact of UGT2B7 His268Tyr polymorphism on the outcome of adjuvant epirubicin treatment in breast cancer. Breast Cancer Res. 13, R57 10.1186/bcr2894 21658222 PMC3218946

[B71] Patt D. A. Duan Z. Fang S. Hortobagyi G. N. Giordano S. H. (2007). Acute myeloid leukemia after adjuvant breast cancer therapy in older women: understanding risk. J. Clin. Oncol. 25, 3871–3876 10.1200/JCO.2007.12.0832 17664457

[B72] Perou C. M. Sørlie T. Eisen M. B. van de Rijn M. Jeffrey S. S. Rees C. A. (2000). Molecular portraits of human breast tumours. Nature 406, 747–752 10.1038/35021093 10963602

[B73] Pinder M. C. Duan Z. Goodwin J. S. Hortobagyi G. N. Giordano S. H. (2007). Congestive heart failure in older women treated with adjuvant anthracycline chemotherapy for breast cancer. J. Clin. Oncol. 25, 3808–3815 10.1200/JCO.2006.10.4976 17664460

[B74] Rae J. M. (2011). Personalized tamoxifen: what is the best way forward? J. Clin. Oncol. 29, 3206–3208 10.1200/JCO.2011.39.0971 21768456

[B75] Rae J. M. Drury S. Hayes D. F. Stearns V. Thibert J. N. Haynes B. P. (2011). Lack of correlation between gene variants in tamoxifen metabolizing enzymes with primary endpoints in the ATAC trial. Cancer Res. 70, S1–S7

[B76] Rae J. M. Sikora M. J. Henry N. L. Li L. Kim S. Oesterreich S. (2009). Cytochrome P450 2D6 activity predicts discontinuation of tamoxifen therapy in breast cancer patients. Pharmacogenomics J. 9, 258–264 10.1038/tpj.2009.14 19421167 PMC2991048

[B77] Regan M. M. Leyland-Jones B. Bouzyk M. Pagani O. Tang W. Kammler R. (2012). CYP2D6 genotype and tamoxifen response in postmenopausal women with endocrine-responsive breast cancer: the breast international group 1-98 trial. J. Natl. Cancer Inst. 104, 441–451 10.1093/jnci/djs125 22395644 PMC3309132

[B78] Rizzo R. Spaggiari F. Indelli M. Lelli G. Baricordi O. R. Rimessi P. (2010). Association of CYP1B1 with hypersensitivity induced by taxane therapy in breast cancer patients. Breast Cancer Res. 124, 593–598 10.1007/s10549-010-1034-5 20632082

[B79] Robertson D. W. Katzenellenbogen J. A. Long D. J. Rorke E. A. Katzenellenbogen B. S. (1982). Tamoxifen antiestrogens. A comparison of the activity, pharmacokinetics, and metabolic activation of the cis and trans isomers of tamoxifen. J. Steroid Biochem. 16, 1–13 10.1016/0022-4731(82)90137-6 7062732

[B80] Ruiter R. Bijl M. J. van Schaik R. H. N. Berns E. M. J. J. Hofman A. Coebergh J.-W. W. (2010). CYP2C19*2 polymorphism is associated with increased survival in breast cancer patients using tamoxifen. Pharmacogenomics 11, 1367–1375 10.2217/pgs.10.112 21047200

[B81] Ruiz C. Tolnay M. Bubendorf L. (2012). Application of personalized medicine to solid tumors: opportunities and challenges. Swiss. Med. Wkly. 142, w13587 22714262 10.4414/smw.2012.13587

[B82] Saadat M. Khalili M. Nasiri M. Rajaei M. Omidvari S. Saadat I. (2012). Clinical response to chemotherapy in locally advanced breast cancer was not associated with several polymorphisms in detoxification enzymes and DNA repair genes. Biochem. Biophys. Res. Commun. 419, 117–119 10.1016/j.bbrc.2012.01.143 22330804

[B83] Schroth W. Goetz M. P. Hamann U. Fasching P. A. Schmidt M. Winter S. (2009). Association between CYP2D6 polymorphisms and outcomes among women with early stage breast cancer treated with tamoxifen. JAMA 302, 1429–1436 10.1001/jama.2009.1420 19809024 PMC3909953

[B84] Schroth W. Hamann U. Fasching P. A. Dauser S. Winter S. Eichelbaum M. (2010). CYP2D6 polymorphisms as predictors of outcome in breast cancer patients treated with tamoxifen: expanded polymorphism coverage improves risk stratification. Clin. Cancer Res. 16, 4468–4477 10.1158/1078-0432.CCR-10-0478 20515869

[B85] Seruga B. Amir E. (2010). Cytochrome P450 2D6 and outcomes of adjuvant tamoxifen therapy: results of a meta-analysis. Breast Cancer Res. Treat. 122, 609–617 10.1007/s10549-010-0902-3 20454926

[B86] Soerjomataram I. Louwman M. W. J. Ribot J. G. Roukema J. A. Coebergh J. W. W. (2008). An overview of prognostic factors for long-term survivors of breast cancer. Breast Cancer Res. Treat. 107, 309–330 10.1007/s10549-007-9556-1 17377838 PMC2217620

[B87] Sørlie T. Perou C. M. Tibshirani R. Aas T. Geisler S. Johnsen H. (2001). Gene expression patterns of breast carcinomas distinguish tumor subclasses with clinical implications. Proc. Natl. Acad. Sci. U.S.A. 98, 10869–10874 10.1073/pnas.191367098 11553815 PMC58566

[B88] Stephens P. J. Tarpey P. S. Davies H. Van Loo P. Greenman C. Wedge D. C. (2012). The landscape of cancer genes and mutational processes in breast cancer. Nature 486, 400–404 22722201 10.1038/nature11017 PMC3428862

[B89] Sweeney C. Ambrosone C. B. Joseph L. Stone A. Hutchins L. F. Kadlubar F. F. (2003). Association between a glutathione S-transferase A1 promoter polymorphism and survival after breast cancer treatment. Int. J. Cancer 103, 810–814 10.1002/ijc.10896 12516103

[B90] Teh L. K. Mohamed N. I. Salleh M. Z. Rohaizak M. Shahrun N. S. Saladina J. J. (2012). The risk of recurrence in breast cancer patients treated with tamoxifen: polymorphisms of CYP2D6 and ABCB1. AAPS J. 14, 52–59 10.1208/s12248-011-9313-6 22183189 PMC3291182

[B91] Toyama T. Yamashita H. Sugiura H. Kondo N. Iwase H. Fujii Y. (2009). No association between CYP2D6*10 genotype and survival of node-negative Japanese breast cancer patients receiving adjuvant tamoxifen treatment. Jpn. J. Clin. Oncol. 39, 651–656 10.1093/jjco/hyp076 19596663

[B92] Tsai S. M. Lin C. Y. Wu S. H. Hou L. A. Ma H. Tsai L. Y. (2009). Side effects after docetaxel treatment in Taiwanese breast cancer patients with CYP3A4, CYP3A5, and ABCB1 gene polymorphisms. Clin. Chim. Acta 404, 160–165 10.1016/j.cca.2009.03.038 19332043

[B93] Tucker A. N. Tkaczuk K. A. Lewis L. M. Tomic D. Lim C. K. Flaws J. A. (2005). Polymorphisms in cytochrome P4503A5 (CYP3A5) may be associated with race and tumor characteristics, but not metabolism and side effects of tamoxifen in breast cancer patients. Cancer Lett. 217, 61–72 10.1016/j.canlet.2004.08.027 15596297

[B94] van Schaik R. H. Kok M. Sweep F. C. van Vliet M. van Fessem M. Meijer-van Gelder M. E. (2011). The CYP2C19*2 genotype predicts tamoxifen treatment outcome in advanced breast cancer patients. Pharmacogenomics 12, 1137–1146 10.2217/pgs.11.54 21830868

[B95] Watson M. A. Stewart R. K. Smith G. B. Massey T. E. Bell D. A. (1998). Human glutathione S-transferase P1 polymorphisms: relationship to lung tissue enzyme activity and population frequency distribution. Carcinogenesis 19, 275–280 10.1093/carcin/19.8.1495 9498276

[B96] Wegman P. Elingarami S. Carstensen J. Stal O. Nordenskjöld B. Wingren S. (2007). Genetic variants of CYP3A5, CYP2D6, SULT1A1, UGT2B15 and tamoxifen response in postmenopausal patients with breast cancer. Breast Cancer Res. 9, R7 10.1186/bcr1713 17244352 PMC1851378

[B97] Wegman P. Vainikka L. Stål O. Nordenskjöld B. Skoog L. Rutqvist L. E. (2005). Genotype of metabolic enzymes and the benefit of tamoxifen in postmenopausal breast cancer patients. Breast Cancer Res. 7, R284–R290 10.1186/bcr1326 15987423 PMC1143572

[B98] World Health Organization (WHO) (2004). The Global Burden of Disease: 2004 Update. Lyon: World Health Organization

[B99] Xu Y. Sun Y. Yao L. Shi L. Wu Y. Ouyang T. (2008). Association between CYP2D6*10 genotype and survival of breast cancer patients receiving tamoxifen treatment. Ann. Oncol. 19, 1423–1429 10.1093/annonc/mdn155 18407954

[B100] Yao S. Barlow W. E. Albain K. S. Choi J. Y. Zhao H. Livingston R. B. (2010). Gene polymorphisms in cyclophosphamide metabolism pathway, treatment-related toxicity, and disease-free survival in SWOG 8897 clinical trial for breast cancer. Clin. Cancer Res. 16, 6169–6176 10.1158/1078-0432.CCR-10-0281 21169260 PMC3058716

[B101] Zárate R. González-Santigo S. de la Haba J. Bandres E. Morales R. Salgado J. (2007). GSTP1 and MTHFR polymorphisms are related with toxicity in breast cancer adjuvant anthracycline-based treatment. Curr. Drug Metab. 8, 481–486 10.2174/138920007780866780 17584018

[B102] Zhang B. L. Sun T. Zhang B. N. Zheng S. Lü N. Xu B. H. (2011). Polymorphisms of GSTP1 is associated with differences of chemotherapy response and toxicity in breast cancer. Chin. Med. J. 124, 199–204 21362365

